# Transcriptional analysis of primary ciliary dyskinesia airway cells reveals a dedicated cilia glutathione pathway

**DOI:** 10.1172/jci.insight.180198

**Published:** 2024-07-23

**Authors:** Jeffrey R. Koenitzer, Deepesh Kumar Gupta, Wang Kyaw Twan, Huihui Xu, Nicholas Hadas, Finn J. Hawkins, Mary Lou Beermann, Gervette M. Penny, Nathan T. Wamsley, Andrew Berical, Michael B. Major, Susan K. Dutcher, Steven L. Brody, Amjad Horani

**Affiliations:** 1 Department of Medicine and; 2 Department of Pediatrics, Washington University School of Medicine, St. Louis, Missouri, USA.; 3 Center for Regenerative Medicine and; 4 The Pulmonary Center, Department of Medicine, Boston University and Boston Medical Center, Boston, Massachusetts, USA.; 5 Department of Genetics and; 6 Department of Cell Biology and Physiology, Washington University School of Medicine, St. Louis, Missouri, USA.

**Keywords:** Cell biology, Pulmonology, Cell stress, Genetic diseases, iPS cells

## Abstract

Primary ciliary dyskinesia (PCD) is a genetic condition that results in dysmotile cilia. The repercussions of cilia dysmotility and gene variants on the multiciliated cell remain poorly understood. We used single-cell RNA-Seq, proteomics, and advanced microscopy to compare primary culture epithelial cells from patients with PCD, their heterozygous mothers, and healthy individuals, and we induced pluripotent stem cells (iPScs) generated from a patient with PCD. Transcriptomic analysis revealed unique signatures in PCD airway cells compared with their mothers’ cells and the cells of healthy individuals. Gene expression in heterozygous mothers’ cells diverged from both control and PCD cells, marked by increased inflammatory and cellular stress signatures. Primary and iPS-derived PCD multiciliated cells had increased expression of glutathione-S-transferases *GSTA2* and *GSTA1*, as well as NRF2 target genes, accompanied by elevated levels of reactive oxygen species (ROS). Immunogold labeling in human cilia and proteomic analysis of the ciliated organism *Chlamydomonas reinhardtii* demonstrated that GSTA2 localizes to motile cilia. Loss of human GSTA2 and *C*. *reinhardtii* GSTA resulted in slowed cilia motility, pointing to local cilia regulatory roles. Our findings identify cellular responses unique to PCD variants and independent of environmental stress and uncover a dedicated ciliary GSTA2 pathway essential for normal motility that may be a therapeutic target.

## Abstract 2

Single cell RNA sequencing analysis of ciliated airway cells from primary ciliary dyskinesia subjects reveals widespread transcriptional changes and identifies cilia dedicated protective mechanisms.

## Introduction

Motile cilia in the airways are a site of first encounter with the environment and are essential for clearing pathogens and foreign particulates (1, 2). Dysfunction of motile cilia occurs in primary ciliary dyskinesia (PCD), a largely autosomal recessive syndrome caused by pathogenic variants in over 50 cilia-associated genes that has no specific treatments. PCD is characterized by chronic sinopulmonary infection, infertility, and abnormal organ laterality (1, 3–5). Individuals who are heterozygous for pathogenic variants (carriers) have no clinical disease, with the exception of variants in *FOXJ1* (6). Progress toward therapies is slow and it is now clear that PCD variants result in abnormal or absent cilia motility. However, the identification of underlying mechanisms and molecular repercussions of the variants have received little attention.

Transcriptional profiling of airway epithelial cells has been an indispensable approach to uncover pathogenic pathways in asthma, chronic obstructive pulmonary disease (COPD), and cystic fibrosis (CF), revealing unique cellular compositions in disease and identifying potential targets for therapy (7–9). In CF, also a genetic condition associated with lung disease and abnormal ciliary-mucus interactions, cellular stress due to the CFTR F508del variant (in the absence of infection) is proposed to contribute to lung disease progression (10, 11). We have previously shown that variants in PCD genes (*DNAAF5*, *SPAG1*, and *DNAAF2*) have elevated levels of the stress-related protein SQSTM1/p62 (12). However, it is unclear if the molecular effect on cells is the result of the airway environment or the specific genetic variant.

To address the effects of PCD variants on airway epithelium homeostasis, we employed single cell RNA-Seq (scRNAseq) to characterize the expression profile of PCD airways, specifically the multiciliated cells. We initially investigated patients with variants in *DNAH5*. DNAH5 is a dynein axonemal motor protein that is uniquely expressed in motile cilia. Variants in *DNAH5* are among the most common causes of PCD and typically cause paralyzed cilia owing to the absence of the outer dynein arm complex, which contains the major molecular motors that propel cilia beating (13–15). To separate the effects of the genetic variants from those caused by differences induced by the airway’s environment (e.g., local inflammation, infection, and exposure), primary nasal cells were harvested from the airways and first cultured in vitro before analysis. We compared PCD patient-derived cells (PCD cells) to those obtained from their healthy heterozygous mothers and healthy unrelated volunteers. Results were further confirmed in analysis of induced pluripotent stem cells (iPScs) (16) derived airway cells from a patient and a healthy control.

Single cell transcriptional profiling of primary culture airway cells identified conservation of the multiciliated subclusters among the study groups. However, expression patterns in PCD cells differed from their mothers and healthy controls. Notably, PCD cells had altered expression of cilia-related genes and increased expression of glutathione-S-transferases (GSTs), accompanied by an extensive group of other NRF2 (NFE2L2) target genes, suggesting that the genetic variants activate the cellular oxidative stress response pathways. These changes were confirmed in a second set of PCD cells and were conserved in the iPScs PCD cells, indicating that the observed differentially expressed genes were cell intrinsic rather than responses to a chronically infected environment. Analysis of the GST pathway in multiciliated cells uncovered prominent, restricted expression of GSTA2 in motile cilia, which was required for maintenance of normal ciliary motility and was upregulated in PCD cells.

## Results

### PCD variants in DNAH5 do not affect airway cell differentiation fate.

**Figure 1:**
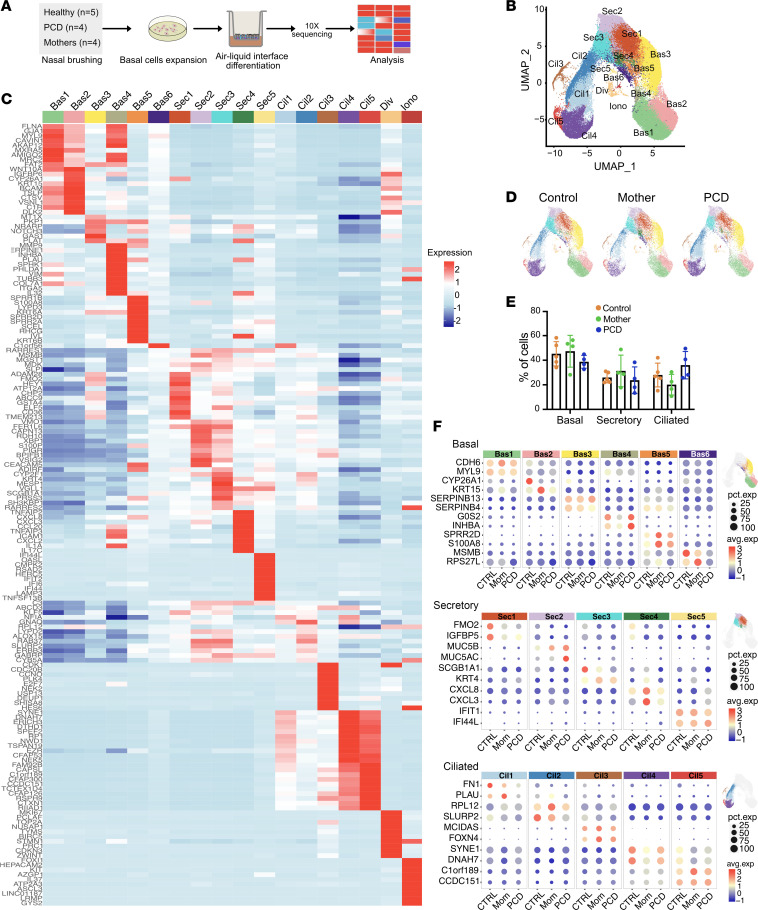
Primary airway cell clustering. (**A**) Schematic of showing nasal cell brush biopsy, culture, and analysis. (**B**) UMAP for dimension reduction of cultured nasal cells showing unsupervised cell clustering and distribution of basal (Bas), secretory (Sec), multiciliated (Cil), dividing (Div), and ionocytes (Ion) (*n* = 5 samples, approximately 7,000 cells/sample). Colors represent different subclusters. (**C**) Heatmap of identified cell clusters shown in the UMAP, showing top expressed genes per cell subcluster. Unique transcriptional signatures differentiated the different subclusters. (**D**) UMAPs comparing cell distribution between normal control cells, heterozygous *DNAH5* mothers, and homozygous *DNAH5* patients’ cells show no differences in cell subclusters between groups (*n* = 5 for normal cells, *n* = 4 for heterozygous, and *n* = 4 PCD cells). (**E**) Cell numbers of the major cell types comparing the different experimental groups. (**F**) Dotplot comparing the different cell subclusters of each submitted sample between the indicated experimental groups.

To link transcriptional profiles to specific cell types in PCD cells, we performed scRNAseq in airway cells from 4 unrelated patients with pathogenetic variants in *DNAH5* (Figure 1A and Supplemental Table 1; supplemental material available online with this article; https://doi.org/10.1172/jci.insight.180198DS1.). All patients were confirmed to have PCD using established genetic and clinical criteria (17) (Supplemental Table 1). For comparison, we obtained nasal cells from 5 unrelated healthy individuals who did not carry pathogenic alleles in 53 known PCD genes, confirmed by whole exome sequencing. As additional controls, we also obtained cells from the heterozygous mother of each participant with PCD.

The airway has a complex interplay between epithelial cells, the local immune cells, and the microbiome of the upper airway. Our goal was to identify epithelial cell responses to the gene variant and motile cilia dysfunction rather than the response to the local environment. Therefore, nasal cells were first expanded in culture at least 10-fold in medium containing antibiotics and antifungal drugs for up to 10 days to minimize the acute effects of the local airway environment. Cells were then differentiated into secretory and multiciliated cells using air-liquid interface (ALI) conditions for another 28 days (18) (Supplemental Figure 1A). Cultured PCD cells showed no cilia motility (as expected) compared with the presence of cilia activity in their heterozygous mothers and healthy controls (Supplemental Figure 1B).

Unsupervised scRNAseq clustering and uniform manifold approximation and projection (UMAP) identified expected airway epithelial cell types as defined by known marker genes, including basal (Bas), secretory (Sec), and multiciliated cells (Cil), as well as a small population of ionocytes (Iono) and dividing cells (Div) (Figure 1B). Within each cell type, multiple subclusters were identified with distinct markers (Figure 1C). These subclusters likely represent different stages of cell differentiation yet show differences in gene expression. There were no significant differences in cell numbers within each cell type (basal, secretory, ciliated) among samples from patients, their heterozygous parents, or controls (Figure 1, D and E, and Supplemental Figure 1C), indicating that the general program of airway differentiation are conserved in PCD *DNAH5* variants. Examining the top markers per cell cluster and considering each of the sequenced samples, we found that the clusters’ identities were broadly preserved among the different samples (Figure 1F).

### Transcriptional analysis identifies unique subcell clusters of ciliated cells.

**Figure 2:**
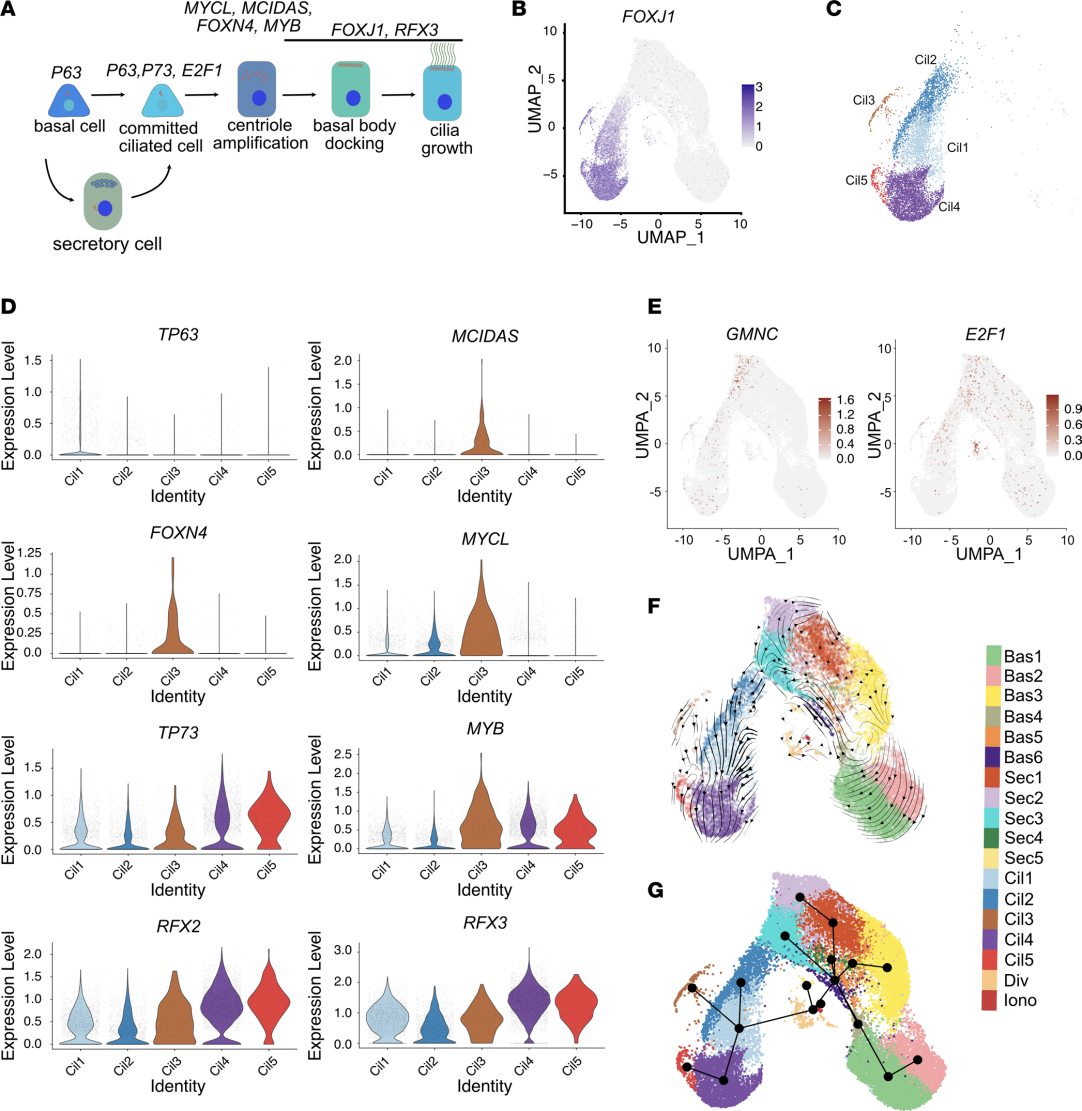
Multiciliated cells single cell analysis and velocity trajectories. (**A**) Scheme depicting the canonical differentiation pathway of multiciliated cells. (**B**) *FOXJ1*, the ciliogenesis master regulator, is shown overlayed on the scRNAseq UMAP to show the multiciliated cell clusters. (**C**) UMAP of extracted *FOXJ1^+^* cells shows 5 unique multiciliated cell subclusters, marked Cil1 for (FOXJ1^+^ P63^+^) basalociliated cells, Cil2 for (FOXJ1^+^ SLURP2^+^) secretociliated cells, Cil3 for (FOXJ1^+^ CCNO^+^ DEUP1^+^ PLK4^+^) deuterostomal cells, Cil4 for (FOXJ1^+^ DNAH7^+^, low c1orf189) early mature multiciliated cells, and Cil5 for (FOXJ1^+^ DNAH7^+^, high c1orf189) late mature multiciliated cells. (**D**) Violin plot showing known multiciliated cell differentiation transcriptional markers in each of the multiciliated cell subclusters. (**E**) UMAP showing expression levels of *GMNC* and *E2F1* expression levels in airway cells. (**F**) Pseudotime analysis showing projected differentiation trajectory of cells overlayed on the UMAP of cell clusters showing no trajectories from secretory cells into ciliated cells. (**G**) Velocity analysis showing unsupervised high-dimensional vectors predicting future state of individual cells, demonstrating a direct relationship between differentiation of basal cells and multiciliated cells.

To identify potential differences among our study groups and the impact of the *DNAH5* allele, we further analyzed the subclusters of multiciliated cell (Cil1–Cil5). We first determined if the cell subclusters can be defined by the known sequence of transcription factors expression and their relationship to the multiciliated cell assembly pathway (19) (Figure 2A). We defined multiciliated cells by the expression of the transcription factor *FOXJ1* (Figure 2B). Unsupervised clustering identified 5 subclusters of multiciliated cells that could be distinguished by the pattern of expression of differentially expressed genes (Figure 1C). Using known transcription factors and motile cilia markers, we defined these subclusters as “basalociliated cells” (*FOXJ1^+^* *P63^+^*) (Cil1), “secretociliated cells” (*FOXJ1^+^ SLURP2^+^*) (Cil2), “deuterostomal cells” (*FOXJ1^+^ CCNO^+^* *DEUP1^+^* *PLK4^+^*) (Cil3), early mature ciliated cells (*FOXJ1^+^ DNAH7^+^*, low *CFAP141(c1ORF189)*) (Cil4), and late mature ciliated cells (*FOXJ1^+^ DNAH7^+^*, high *CFAP141*) (Cil5) (Figure 2C and Figure 1C). Several microtubule inner protein (MIP) genes were highly expressed in late mature ciliated cells (Cil5) compared with early mature ciliated cells (Cil4) subclusters, including *CFAP276* (*C1ORF194*), *CIMIP1*(*C20ORF85*), and *CFAP141*(*C1ORF189*). Several MIP proteins are found in the lumen of axonemal microtubules and are thought to organize the periodicity of the ciliary motors and their regulator components (20), which may suggest their requirement at later stages of cilia assembly.

Analysis of the expression levels of defined ciliogenesis transcription factors suggested a sequential transition from Cil1 to Cil5 (Figure 2, D and E). Cil1 subcluster had a higher expression of the basal cell marker *TP63*. Moreover, there was a pattern of increasing expression of *MYCL* between Cil1 and Cil3, a transcription factor that was recently shown to be active at the branch point of basal to multiciliated cell fate (21). Pseudotime and RNA velocity analysis of all clusters indicated that a high percentage of multiciliated cells likely differentiate directly from basal progenitor cells through an intermediary dividing cell cluster (Div) without transitioning through secretory cells (Figure 2, F and G) (21). Multiciliated cells with homozygous variants in *DNAH5* did not use different pathways of differentiation compared with the other groups.

### DNAH5 variant airway cells have unique gene expression compared with the other groups.

**Figure 3:**
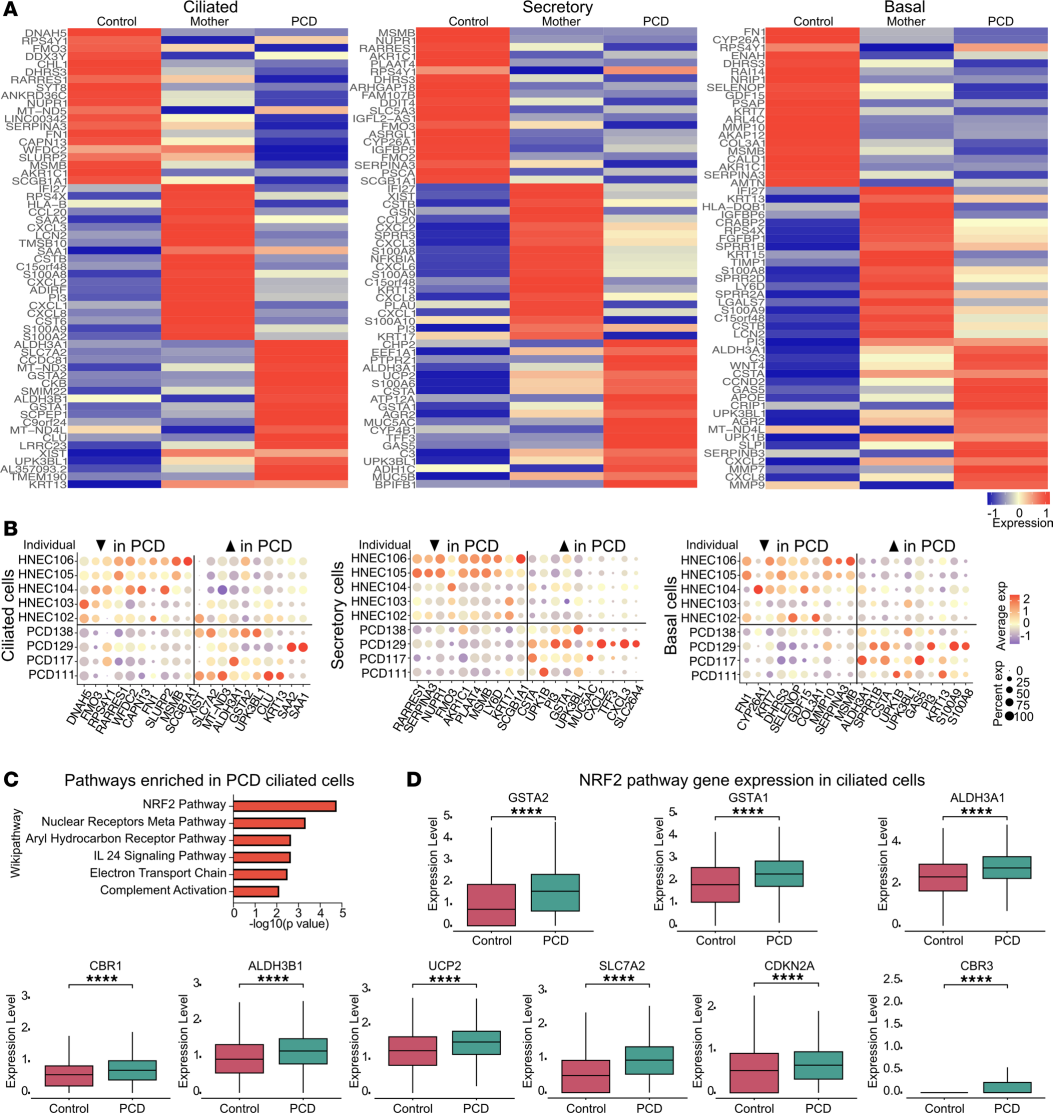
scRNAseq comparison between PCD and non-PCD cells. (**A**) Heat map showing the top differentially expressed genes in ciliated, secretory, and basal cells of PCD patient primary nasal cells compared with heterozygous maternal cells and healthy control cells demonstrating unique differences among the different groups. (**B**) Dotplot depicting the transcript expression levels of all samples analyzed in **A** showing the highest differentially expressed genes per sample. (**C**) Pathway analysis of the top differentially expressed genes in multiciliated cells, comparing cells from patients with PCD to cells from people in the healthy control group. (**D**) Box plots showing the differential gene expression in multiciliated cells of select NRF2 pathway genes between PCD and healthy control cells. In panels **A**, **B**, and **C**, *n* = 5 control samples, *n* = 4 heterozygous, and *n* = 4 PCD samples. In panel **D**, *n* = 5 control samples and *n* = 4 PCD samples. 3 technical replicates each. ****P < 0.0001. Error bars represent standard deviation unless noted otherwise.

To identify pathways that are perturbed as the result of variants in *DNAH5*, we compared the differential gene expression among the 3 groups of cultured airway cells using unsupervised analysis. Differential gene expression was identified in all major cell types among PCD, maternal, and control cells (Supplemental Figure 2A). Examining the most differentially expressed genes between groups, we found that differences between PCD cells and control cells indicated a clear distinction (Figure 3A), with a high degree of concordance among different individuals with PCD (Figure 3B). Since our control samples had a male bias, we performed a subanalysis between the different groups, using samples from women only (including additional control samples from published data sets from women (22, 23), identifying similar results (Supplemental Figure 2B).

Examining the top upregulated genes in multiciliated PCD cells compared with control cells identified genes related to cilia, inflammation, and downstream effectors of the NRF2 oxidative stress response pathway (24, 25) (Figure 3C and Supplemental Table 2). The latter included NRF2-activated genes *SLC7A2*, *ALDH3A1*, *GSTA1*, and *GSTA2*, with *GSTA1* and *GSTA2* being among the highest differentially expressed genes in multiciliated cells (Figure 3D). Although *DNAH5* expression is unique to multiciliated cells, secretory and basal cells also contributed to the group of differentially expressed genes between variant and control cells (Figure 3A). In secretory and basal cells, gene ontology terms and pathways associated with genes upregulated in PCD cells suggested induction of inflammation-related signaling. Compared with control basal and secretory cells, PCD cells had a decrease in TGF-β–related genes, suggesting that the roles of this pathway may be dysregulated in PCD (Supplemental Figure 2, C and D).

To confirm these results, we independently harvested and cultured primary nasal cells from additional individuals with PCD and individuals who were healthy and analyzed differential gene expression using bulk RNAseq. Specifically, primary culture nasal cells from patients with PCD homozygous for 2 additional but different homozygous pathogenic variants in *DNAH5* and 1 in *HYDIN* were compared with cells from healthy individuals. Cells were cultured in vitro and allowed to redifferentiate into ciliated and nonciliated cells using ALI conditions before analysis (18). Like the scRNAseq analysis, bulk RNAseq analysis of these cultures also showed that genes related to NRF2 and glutathione-related pathways were enriched in the PCD cells compared with normal cells (Supplemental Table 3 and Supplemental Figure 3) (26).

### Cells from mothers’ heterozygous variants in DNAH5 show transcriptional divergence.

**Figure 4:**
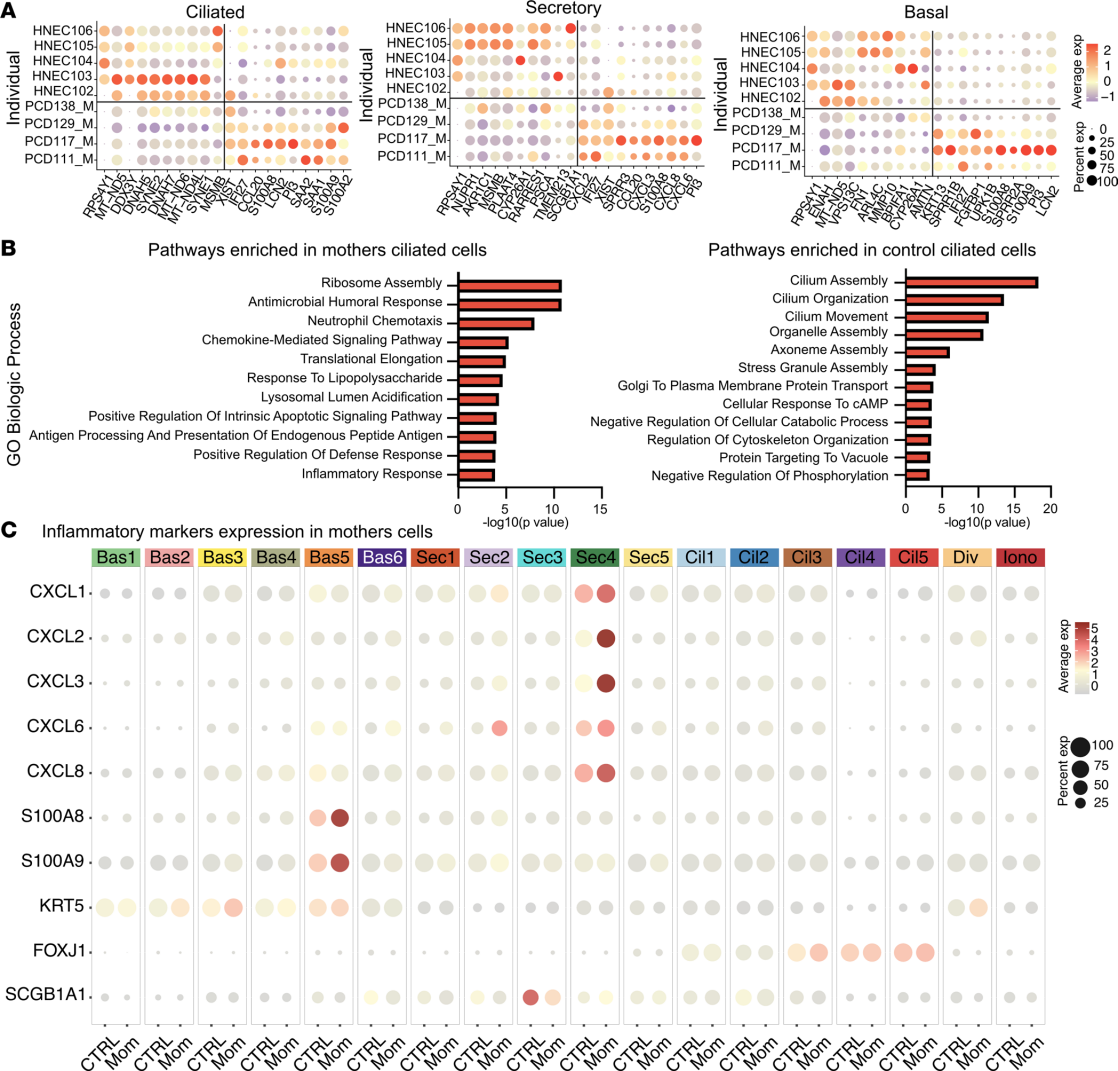
Differential gene expression of heterozygous maternal cells. (**A**) Dotplot showing the relative differential gene expression between heterozygous cells and healthy control cells without variants in known PCD genes. (**B**) Pathway analysis of differentially expressed genes in heterozygous *DNAH5* multiciliated cells compared with control cells. (**C**) Dotplot showing differentially expressed inflammatory markers between heterozygous maternal and control cells within the different cell subclusters. *n* = 5 control samples and *n* = 4 heterozygous samples.

Unexpectedly, we found that the transcriptome of the heterozygous mothers cells differed from both control and PCD cells (Figure 3A). We observed differential gene expression in all major cell types in cultures of the 4 unrelated mothers with heterozygous *DNAH5* compared with healthy individuals who had no pathogenetic variants in known PCD-associated genes (Figure 3A and Figure 4A). Differences in the expression profile of mothers’ heterozygous cells were also observed when only female samples from control and PCD cultures were included in the analysis (including additional control samples from women in publicly available data sets) (Supplemental Figure 2B). Interestingly, the gene expression profile of heterozygous cells was closer to PCD cells than to control non-PCD cells.

Examining the molecular pathways driven by the transcriptional changes in the mothers’ heterozygous cells compared with the control cells showed increased expression of genes related to inflammation and cytokine production (Figure 4B and Supplemental Figure 4). Inflammatory markers were found in all cell types but were particularly enriched in clusters Sec 4 and Bas 5 (Figure 4C), which may be due to effects of a cilia-related dysfunction on neighboring cells.

### PCD cells have increased cellular stress markers.

**Figure 5:**
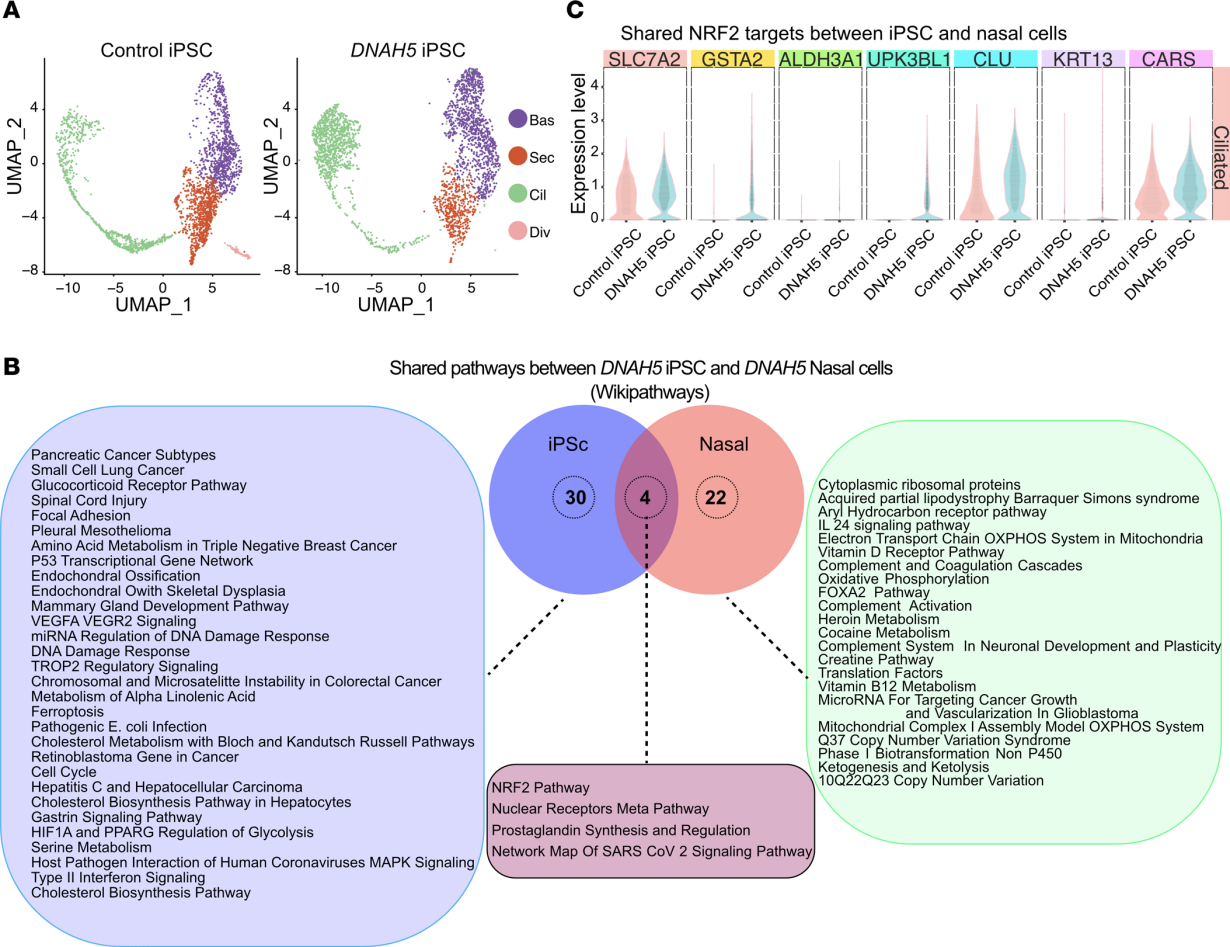
Expression of GSTA2 and NRF2 markers in native and *DNAH5* iPScs. (**A**) UMAPs comparing cell distribution between control iPS and *DNAH5* iPScs showing no differences in cell subclusters between groups. Different colors represent different cell types. (1 donor, *n* = 3 technical replicates combined). (**B**) Pathway analysis of differentially expressed genes comparing nasal PCD cells and iPS PCD cells generated from the same patient with PCD. Diagram shows unique pathways upregulated in iPS PCD cells compared with control iPS cells, those upregulated in the nasal PCD cells compared with control nasal cells, and pathways that are shared between the iPS PCD cells and the nasal PCD cells (*n* = 1 donor for iPS PCD cells, *n* = 1 donor for control iPS cells, *n* = 1 donor for nasal PCD cells, *n* = 5 control nasal cells. Each in *n* = 3 technical replicates combined). (**C**) Violin plots showing the differential gene expression of select NRF2 pathway genes shared between multiciliated cells originating from induced pluripotent cells and native nasal PCD cells.

We considered that the differential gene expression and induction of the NRF2 pathway observed in multiciliated PCD cells could be either cell intrinsic due to the PCD variant or cell extrinsic due to changes induced by the airway inflamed environment. At the onset, we chose to examine the differential gene expression in cultured cells rather than freshly harvested cells to separate these effects. To further examine the consequence of the genetic variant, we generated iPScs from the peripheral leukocytes of one of the patients with PCD (16). Unlike the primary nasal PCD cells, the iPScs were never exposed to the airway environment. These cells were also differentiated to multiciliated cells using ALI conditions and underwent transcriptional analysis using scRNAseq. Unsupervised integrated analysis categorized all the major epithelial cell types (Figure 5A), including *KRT5^+^* basal cells (Bas), *SLURP2^+^/FOXJ1^–^/KRT5^–^* secretory cells (Sec), *FOXJ1^+^* multiciliated cells (Cil), and a small dividing cell population (Div). Ionocytes were not identified in these cells. As with primary airway cells, the cell type proportions were similar between *DNAH5* iPScs and control iPScs.

The differences in gene expression between the multiciliated cell clusters from *DNAH5* iPScs and control iPScs mirrored the changes observed in the primary nasal cells (Figure 5, B and C). To confirm changes in NRF2 target genes identified in PCD nasal cells, we compared the expression levels of these targets in the *DNAH5* iPScs (Figure 5C). We found increased expression of genes related to oxidative stress, including increased expression of *GSTA1* and *GSTA2* (Figure 5C). These results indicate that the increased expression of pathways related to cellular stress, especially NRF2 downstream effectors, are cell intrinsic and due to a direct effect of the *DNAH5* variants. This indicates that the gene expression changes are less likely to be caused by environment due to an infected or inflamed airway in the patients’ nasal passages.

### GSTs and NRF2 pathway transcripts are increased in PCD.

**Figure 6:**
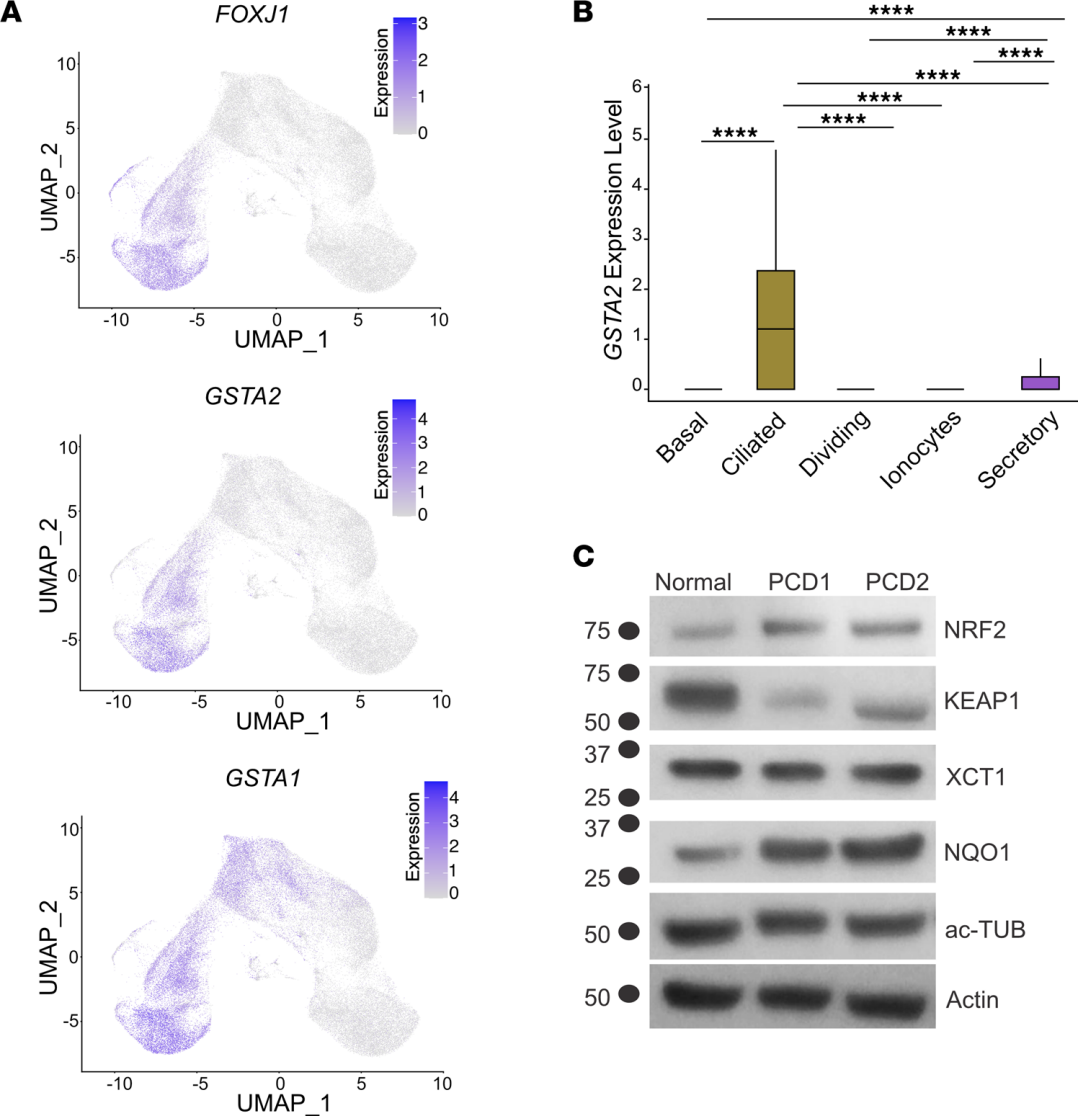
Expression of GSTA2 and NRF2 markers in multiciliated cells. (**A**) The expression levels of *GSTA1* and *GSTA2* projected on UMAPs of airway cells compared with the expression of *FOXJ1* as a marker of multiciliated cells. (**B**) Median expression of *GSTA2* in airway cells. (**C**) Immunoblot of NRF2 pathway targets in cells from 2 patients with PCD. In **A** and **B**, *n* = 5 normal samples, 3 technical replicates each. ****P < 0.0001, respectively. Error bars represent SD unless noted otherwise.

We focused on *GSTA2* and *GSTA1*, which were among the highest differentially expressed transcripts in PCD multiciliated cells. The *GSTA2* transcripts were highly expressed in multiciliated airway cells while *GSTA1* transcripts were expressed in both multiciliated and secretory cells, as shown by comparing expression overlay of *GSTA1* and *GSTA2* to *FOXJ1* on the UMAP data (Figure 6A). GSTA2 expression was previously reported in multiciliated cells in the fallopian tubes (27) and was suggested to be a specific marker of multiciliated cells (28). However, closer inspection of the mean expression levels of *GSTA2* showed very low transcript levels in secretory cells as well (Figure 6B).

Increased *GSTA1*, *GSTA2*, and NRF2 target gene transcripts in multiciliated PCD cells pointed to a wider cellular mechanism of defense against cellular stress in PCD that includes oxidative and electrophilic stress. Indeed, NQO1 protein levels, a key canonical NRF2 target gene (29), was increased at the protein levels in PCD cells (Figure 6C). To confirm these results, we used independent cultures of normal and PCD cells analyzed by a sensitive, targeted mass spectrometry method to identify predefined NRF2 targets (Optimized internal standard–triggered parallel reaction monitoring, OIS-PRM) (30–32). OIS-PRM uses a panel of proteins that are known NRF2 targets but are not specific to airway epithelial cells and did not include specific probes for GSTA2 or GSTA1. Using OIS-PRM, we detected increased protein levels of several NRF2 targets within the log_2_-fold range considered significant using this technique (Table 1 and Supplemental Table 4) (30, 32). These increased levels paralleled the elevated transcripts levels detected by scRNAseq, confirming the activation of the NRF2 pathway in PCD. Thus, there may be a pattern of gene expression directly related to cilia dysfunction and PCD pathobiology due to the *DNAH5* variant.

### Motile cilia have a dedicated glutathione pathway.

**Figure 7:**
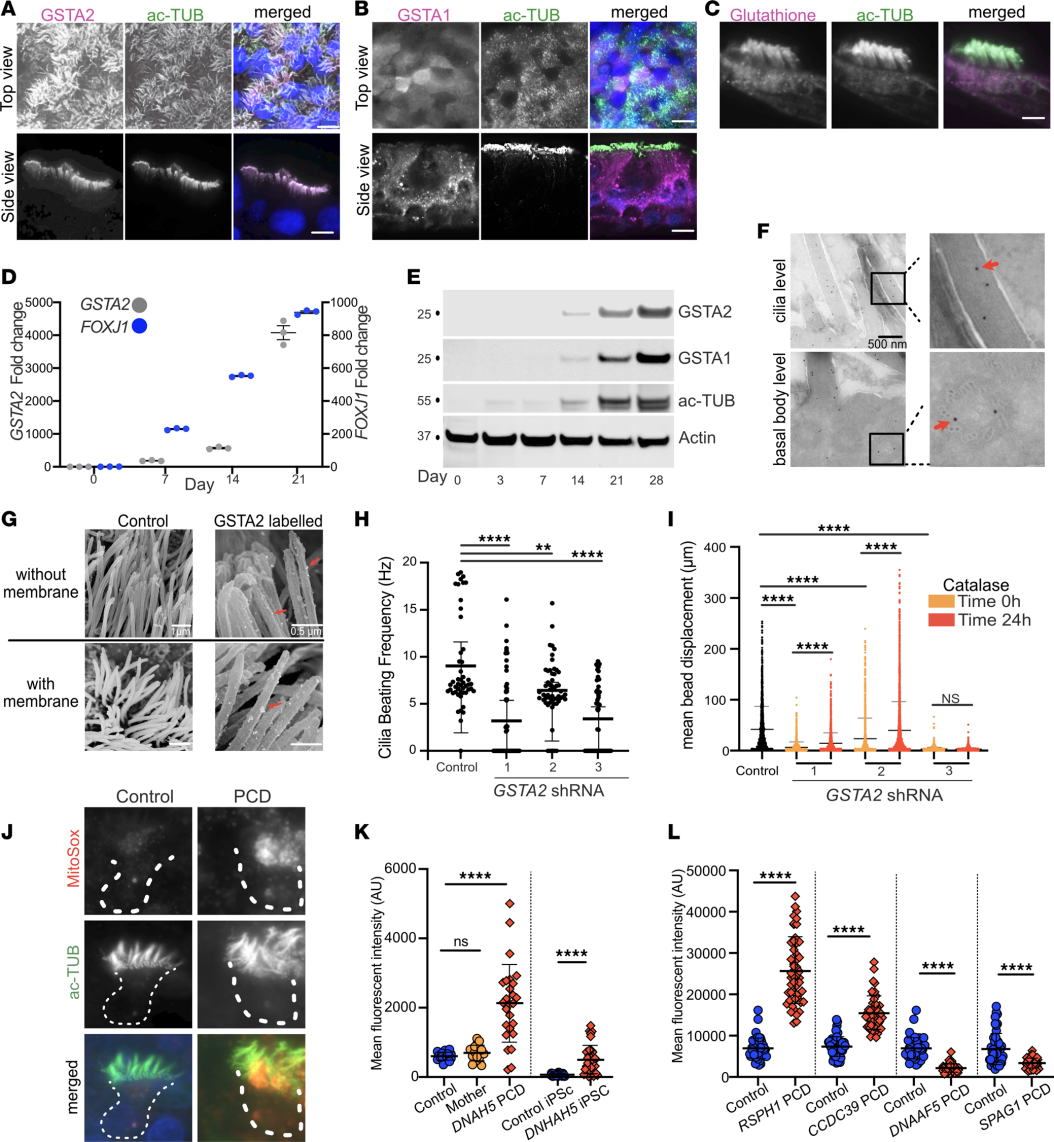
Motile cilia dedicated glutathione pathway. (**A**) Immunofluorescent staining of GSTA2 localization to motile cilia of airway cells. (**B**) Immunofluorescent detection of cytoplasmic GSTA1 in the cytoplasm of multiciliated cells. (**C**) Immunodetection of glutathione in cilia and cytoplasm of multiciliated cells. (**D**) Levels of *GSTA2* and *FOXJ1* during the differentiation of basal cells (ALI day 0) to multiciliated cells, detected by RT-qPCR (*n* = 3 replicates, error bars represent standard error). (**E**) Immunoblot detection of GSTA1 and GSTA2 compared with acetylated α-tubulin (ac-TUB) used as a cilia marker during airway epithelial cell differentiation. (**F**) Immunogold labeling of multiciliated airway cells showing GSTA2 along the cilia axoneme and basal-body microtubules. (**G**) Scanning electron microscopy of multiciliated cells treated with butter to demembranate cilia showing GSTA2 along the cilia microtubules as well as on the ciliary membrane. (**H**) CBF and (**I**) displacement of microbeads across the apical surface of culture primary airway cells following transduction of *GSTA2* shRNA. (**J**) Superoxide levels in multiciliated cells detected by MitoSox. (**K**) MFI of ciliary axonemes showing increased labeling of GSTA2 in PCD and iPS PCD cells compared with control cells (*n* = 3 replicates each). (**L**) MFI of ciliary axonemes showing increased labeling of GSTA2 in *CCDC39* and *RSPH1* PCD cells compared to control cells, and no increase in *DNAAF5* and *SPAG1* PCD cells. In **A**–**L**, *n* = 3 replicates each. Representative images are shown. **P < 0.01, ****P < 0.0001. Error bars represent SD unless noted otherwise. Scale bars = 10μM.

Since *GSTA2* was highly expressed in ciliated cells and was increased in PCD, we determined its cellular localization and relation to motile cilia function. Immunofluorescent staining of normal primary culture nasal cells localized GSTA2 to the motile cilia (Figure 7A and Supplemental Video 1), while GSTA1 was diffusely expressed in the cytoplasm (Figure 7B). To confirm localization of GSTA2 to motile cilia, we expressed either an N- or C-terminal GFP fusion GSTA2 protein in normal airway cells using lentiviral-mediated transduction, driven by a *FOXJ1* promotor. Cells underwent differentiation using ALI conditions and were evaluated for expression of GFP. Live imaging of GFP-GSTA2 transduced cells showed localization of GFP in motile cilia (Supplemental Videos 2 and 3). Moreover, immunofluorescent staining of primary culture airway cells showed localization in motile cilia of tagged GSTA2 when using a GFP antibody (Supplemental Figure 5A). We also found that the substrate of GSTA2, glutathione, was present in the cilia and the cytoplasm (Figure 7C). *GSTA2* mRNA expression increased as multiciliated cells underwent differentiation, showing a temporal relation to ciliogenesis when compared with *FOXJ1* (Figure 7D). Similarly, GSTA2 protein levels increased while more cilia emerged during ciliogenesis, as indicated by the levels of cilia marker acetylated α-tubulin (Figure 7E), further demonstrating a close relationship between GSTA2 expression and motile cilia.

Ciliary GSTA2 suggests a local role in maintaining homeostasis during changes in cilia activity or to protect the axonemes from external damage. To determine whether ciliary GSTA2 localizes to the ciliary microtubules or to the ciliary membrane, we used immunogold-labeled GSTA2 in cultures imaged by electron microscopy. Transmission electron microscopy (TEM) detected labeled GSTA2 along the cilia microtubules (Figure 7F, upper panels) and to a lesser extent along the basal body microtubules (Figure 7F, lower panels).

To determine whether GSTA2 was only restricted to the microtubules, we used scanning electron microscopy (SEM) to image ciliated cells and untreated cells, we used scanning electron microscopy after stripping the cell membranes with a mild detergent. GSTA2 immunogold-labelled cells were compared with those incubated with gold-labeled secondary antibody only (Figure 7G). Like the TEM imaging, the majority of GSTA2-labeling was observed primarily along the ciliary axonemal microtubules (Figure 7G, upper panels) while a smaller fraction was observed on the ciliary membrane (Figure 7G, lower panels).

### GSTA2 is required for maintaining normal cilia motility.

Localization of GSTA2 along the cilia and increased expression in PCD cells strongly suggested a role in motility. To determine a requirement of GSTA2 in normal cilia motility, we knocked down *GSTA2* expression in primary human airway cells using shRNA employing previously described methods (Supplemental Figure 5B and Supplemental Table 5) (33). Compared with nontargeted shRNA sequences, *GSTA2*-specific shRNA sequences reduced ciliary expression of GSTA2 as determined by the mean fluorescent intensity (MFI) of GSTA2 within cilia, which correlated with the degree of decrease in *GSTA2* mRNA (Supplemental Figure 5, B, C, and D).

Cilia function was decreased in the *GSTA2* shRNA treated compared with nontargeted control cells. *GSTA2* knockdown (KD) resulted in a significant decrease in cilia beat frequency (CBF) and ciliary transport of microbeads on the surface of ciliated cells compared with a nontargeted control sequence (Figure 7, H and I). Transport was partially recovered after treating cells with catalase, an enzyme that neutralizes reactive oxygen species. The partial recovery of cilia motility after catalase treatment of *GSTA2* KD confirmed a role for GSTA2 in modulating oxidative burden in ciliated cells, likely within the ciliary axoneme. Since the mitochondria are a major contributor of endogenous reactive species (34), we measured the levels of superoxide activity in cells from a participant with PCD. The MitoSox fluorescent indicator level was higher in live cultured PCD nasal cells compared with control non-PCD cells (Figure 7J).

Like the increase in *GSTA2* transcripts in *DNAH5* variant cells, endogenous GSTA2 levels in the cilia of these cells were increased compared with controls, measured by the MFI of GSTA2 in *DNAH5* PCD cells compared with control cells (Figure 7K and Supplemental Figure 5E). Likewise, the MFI of GSTA2 was higher in iPS *DNAH5* cells compared with control normal iPS cells (Figure 7K and Supplemental Figure 5E).

We hypothesized that the increased level of GSTA2 within cilia is a physiologic response to increased retained activity of dynein motors within the cilia of PCD cells. For example, in the *DNAH5* variant cells, the inner dynein arm motors are intact, and only the outer dynein motor arms are missing. We therefore measured the levels of GSTA2 in the cilia of human PCD cells with other pathogenic variants that have different effects on outer and inner dynein motor complexes, including (a) the assembly factor *DNAAF5* (*HEAT2*) that lacks both outer and inner dynein arm motor complexes (35), (b) pathogenic variants of *CCDC39* that have absent inner dynein motors but retain outer dynein motor complexes (36), and (c) *RSPH1* that retains both inner and outer dynein motor arm complexes but has dysmotile cilia due to a defect in the radial spokes (37). Compared with control cells, *CCDC39* PCD and *RSPH1* PCD cells had a higher GSTA2 MFI within cilia, while *DNAAF5* PCD cells (missing all ciliary motors) had lower GSTA2 MFI (Figure 7L and Supplemental Figure 5E). These data suggest that GSTA2 was responding to compensatory persistent dynein complex motor activity within remaining compartments of the dyskinetic cilia.

### Ciliary GSTA2 is evolutionarily conserved.

To discover if GSTA2 expression was evolutionarily conserved, we analyzed published databases of isolated cilia of the algae *Chlamydomonas reinhardtii* (38, 39) and identified 9 GSTs. Three are orthologs of GSTA (α) (Cre16.g688550, Cre16.g670973, and Cre16.g682725). Three are orthologs of GSTT (θ) (Cre17.g708300, Cre15.g636800, and Cre15.g636750), and Cre02.g142200, which is likely to also be a θ ortholog. Cre17.g742300 and Cre17.g742450 are orthologs of GSTP (π) (Supplemental Figure 6A). Using proteomic analysis of isolated cilia that separate the axonemal and membrane/matrix fraction (M+M) (40), 6 GST species are present in cilia and are predominantly found in the M+M (Supplemental Table 6) as opposed to the axonemal-only fraction of isolated cilia (40). The ratio of axonemal to M+M for the GSTA orthologs ranges from 0.73 to 1 (Supplemental Table 6). These findings suggest that a small fraction of the C*hlamydomonas* GST orthologs binds to the microtubules, while the majority reside in the matrix fraction (41), similar to findings in human cilia.

To confirm findings in PCD cells, we performed proteomic analysis of *Chlamydomonas* mutants. Like human *DNAH5* variants, *Chlamydomonas* outer dynein arm docking complex mutants *oda1* and *oda3* (orthologs of the human genes *ODAD1* and *ODAD3*), lack the outer dynein arms and have decreased ciliary motility (42). Indeed, proteomic analysis of cilia axonemes isolates (without the membrane-matrix fraction) from *oda1* and *oda3* mutants, shows that the numbers of peptides from the outer dynein arms are about 3%–5% of the WT control (Supplemental Table 6). Peptides of only 1 of the 9 GSTs are present in these preparations (Cre17.g742300) since axonemes without the membrane/matrix fraction were prepared. Most GST proteins are likely to be lost during the cilia isolation and processing with nonionic detergent. Interestingly, Cre17.g742300 was increased in the mutants compared with WT. We also identified increased levels of ROS-responsive proteins in the docking complex mutant cilia isolates (43), including 2-cys-peroxiredoxin (PRX2), FAP102, and a thioredoxin (TRX) (Supplemental Table 6).

Moreover, like findings of decreased cilia motility in *GSTA2* KD in human cells, an insertional mutant in the *GSTA* ortholog (Cre16.g682725) in *Chlamydomonas* showed reduced swimming velocity as well as reduced CBF compared with a control strain (Supplemental Figure 6, B and C). These results confirm an evolutionary requirement of GSTA for normal cilia motility.

## Discussion

Cellular responses to dysmotility in multiciliated cells from patients with PCD have not been studied. We asked if environmental factors, including chronic airway infection, or genetic factors can lead to adaptive cellular responses and contribute to disease. We used scRNAseq of airway cells from patients with variants in the dynein heavy chain *DNAH5*, and compared them with their heterozygous mothers and unrelated healthy individuals to uncover remarkable differences in gene expression among these groups. These changes include an evolutionarily conserved antioxidant pathway that is upregulated in PCD. scRNAseq of iPScs with a *DNAH5* variant showed similar changes, suggesting that many of the upregulated related pathways arise from genetic variants rather than the environment.

We found that differentially expressed genes in the PCD cells compared with control cells were related to activation of cell protective mechanisms that include downstream effectors of glutathione and the NRF2 pathways. Both pathways were also upregulated in iPScs generated from a patient with PCD (16), indicating that the activation of these response pathways is due to a direct effect of the variant. We found that a highly regulated NRF2 pathway target, GSTA2, was specific to the motile cilia. The cilia-dedicated GSTA2 expression is evolutionarily conserved based on expression in *Chlamydomonas* and is required for normal cilia motility. Baseline and upregulated levels of GSTA2 play a key role in healthy and diseased cilia, respectively.

GSTs are enzymes that protect cells from toxins and electrophilic compounds, including reactive oxygen species, through direct binding to these compounds or by conjugation to glutathione (44–46). We found localization of GSTA2 in motile cilia of normal cells using antibodies against the native protein and confirmed it by expressing tagged forms of GSTA2 in airway cells and by proteomic analysis of isolated cilia from *Chlamydomonas reinhardtii*. Several factors in *DNAH5* mutants may increase ciliary GSTA2. There is a need for cilia to control both external and intrinsic sources of oxidants. Also, there is evidence that normal cilia have multiple proteins that are oxidant-sensitive so that changes in reactive oxidant species balances lead to changes in CBF (47, 48). Moreover, components of the dynein arms are redox sensitive (49), and several redox regulatory proteins, including thioredoxin domain containing proteins, are present in motile cilia (34). One function of these proteins may be related to a need to balance the oxidant load resulting from the metabolism of the continuous supply of ATP (50). We found that the nonmotile cilia in the *DNAH5* PCD variants have a high level of mitochondrial activity and oxidant production detected by Mitosox. We hypothesize that PCD variants, like *DNAH5* variants, that are missing only a subset of dynein motors, have increased activity of the remaining dynein complexes, leading to increased oxidative burden. We found increased levels of GSTA2 in cilia from cells with variants in *DNAH5*, *CCDC39*, and *RSPH1*, all variants with retained subsets of ciliary dynein arm complexes in cilia but ineffective cilia motility. Consistent with our hypothesis, we observed that cilia from cells with variants in the assembly factors *DNAAF5* and *SPAG1*, which result in absence of all dynein arm motor complexes, did not have increased ciliary GSTA2 levels. We do not know if the activity of the retained motors is elevated in an attempted compensatory response.

Our data also suggest a link between a dedicated glutathione metabolism pathway in the cilia and normal ciliary motility. The ciliary expression of GSTA2 allows localized regulation of redox reactions. We found that complete knock out of *GSTA2* using CRISPR/Cas9 resulted in poor growth of multiciliated cells as ciliogenesis commenced (corresponding to ALI days 7–10) with expansion of basal cell clones that did not have efficient *GSTA2* knockouts. This observation may suggest that GSTA2 even contributes to early multiciliated cell function. Partial loss of *GSTA2* in vitro using shRNA KDs results in slowed CBF and reduced transport, indicative of a role of GSTA2 in normal cilia function. Moreover, cilia function partially recovers in GSTA2 knockdown after treatment with catalase, which neutralizes reactive oxygen species. These data are further confirmed by similar effects on cilia motility in mutants of *gst* (Cre16.g682725) in *Chlamydomonas*. Further, we have found that 6 redox-active proteins that were previously identified in *Chlamydomonas* (51) were increased in the *Chlamydomonas* outer arm docking complex mutants. The *Chlamydomonas* Cre16.g682725 *gst* was previously shown to be upregulated by stress conditions created by singlet oxygen (52). All together, these data suggest an upregulation of antioxidant pathways in an attempt to compensate for elevated redox activity.

The increased GSTA2 protein in motile cilia from PCD cells is likely part of a generalized cellular response, as evidenced by increased expression of other NRF2 downstream effectors in the PCD variants. The NRF2 pathway is a key transcriptional pathway that regulates the expression of factors for detoxification, antioxidation, and stress responses. NRF2 targets include the broad expression of antioxidants and genes regulating metabolic control for the protective response (24). The importance of this pathway in airway cell protection was previously shown in acute lung infection, COPD, and exposure to cigarette smoke, likely in response to extracellular stimuli (53–55). However, its importance in PCD and its role in maintaining cilia function is unexplored. Our data show a unique transcriptional profile in cultured cells from patients with PCD and reveal increased expression of downstream effectors of the NRF2 pathway. We previously reported formation of SQSTM1/p62 aggregates within airway cells of patients with PCD (12), which may modulate the NRF2 pathway (56). The exact role of GSTA2 within cilia and the NRF2 program in modulating cilia function will require future studies.

Several of the differentially expressed genes in the PCD airway cells were related to inflammatory pathways. This is contrary to a previous report of scRNAseq of a single patient with *DNAH5* PCD who showed reduced expression in immune-related pathways in organoids (57). These differences may be related to the variants in DNAH5 that were studied, influenced by the culture system used, or affected by the degree of differentiation of the cultured cells at the time of analysis.

PCD due to *DNAH5* variants is an autosomal recessive disease. As part of our analysis, we used cells collected from the mothers of the patients with PCD as additional controls. We were surprised to find that mothers who carry a single pathogenic allele (heterozygosity) in *DNAH5* showed a unique transcriptional profile that was distinct form controls and their children with PCD. Heterozygous cells had normal CBF (Supplemental Figure 1B). Heterozygosity in diseases caused by autosomal recessive conditions, as in CF, has been proposed to be associated with limited clinical effects (e.g., infertility) or to modify the phenotype of respiratory conditions that include COPD (58, 59). Heterozygous PCD cells show increased expression of inflammatory markers in nearly all cell types, but most notably in secretory cells (Figure 4C). The causes of differential expression in maternal cells compared with control cells are unclear and will require evaluation of larger cohorts of PCD samples to determine if effects of heterozygosity impact cellular responses. These transcriptional changes may be related to the effects of a subtle cilia dysfunction on neighboring secretory cells. Alternatively, it may also be related to environmental factors and driven by shared pathogens in the airways of parents and their children or from exposure to their chronically infected children. Moreover, these differences may be related to differences in sex. Analysis of lungs of female mice compared with males showed increased expression of inflammatory markers including CXCL2 (60).

scRNAseq analysis is a powerful tool to identify new cell identities and relate differential gene expression differences to a specific cell type. However, DEG testing in scRNAseq carries a risk of false positivity due to pseudoreplication bias (61, 62). We validated our findings using bulk RNAseq experiments from primary nasal cells that were obtained and cultured separately from 3 patients with PCD with known variants affecting cilia motility. Two of the 3 patients were not included in our scRNAseq studies. Identifying activation of the NRF2 and glutathione pathways in these separate experiments further support a potential role of these pathways in PCD (Supplemental Figure 3).

PCD is a rare disease, and we were limited by the availability of samples. Our patient samples included a younger cohort than the control group (patient ages were 4 years to 18 years compared with participants in the control group who were in their 20s to 60s). Prior analysis comparing cells obtained from young children (median age of 5 years) compared with adults suggested changes related to the number of ciliated and mucus cells between groups, and increased expression of genes related to cilia, mitochondria, and oxidative phosphorylation in older individuals (63). We have not observed changes in developmental pathways in our analysis.

In summary, our data using primary airway cells from patients with PCD and confirmed using iPScs PCD cells and the model organism *Chlamydomonas*, provide insight into involvement of the NRF2 pathway in PCD and show that GSTA2, a GST, is a novel motile cilia protein. Our findings indicate that motile cilia have a dedicated glutathione pathway that is required for normal cilia motility and is upregulated in motile cilia disease. These data show that variants affecting cilia motility result in widespread transcriptional changes and identify cilia-dedicated protective mechanisms essential for normal cilia function and independent of environmental stress.

## Methods

### Sex as a biological variable

We considered sex as a biological variable and obtained cells from patients and healthy individuals of both sexes. Since PCD is a rare genetic condition, we were limited by the availability of individuals carrying pathogenic variants in the PCD genes under study. Heterozygous maternal cells are all from female participants, which may affect comparisons with control cells, which had a relative over representation of male participants. To account for the male bias in our control samples, we supplemented our analysis with female control samples from published data sets and performed additional comparisons between female-only samples (22, 23).

### Participants

Individuals with known pathogenic variants causative of PCD and clinical diagnosis of PCD were recruited from the PCD and Rare Airway Disease clinic at St. Louis Children’s Hospital and Washington University after informed consent was obtained from individuals (or their legal guardians).

### Airway epithelia cell culture

Deidentified nasal epithelial cells were isolated by biopsy of the inferior nasal turbinate performed using a cytology brush. Primary airway cells were processed, expanded in culture, and then differentiated using ALI conditions in Transwell (Corning Inc.) supports as previously described (12, 18). iPS cells were generated, differentiated into airway epithelial cells, and cultured at ALI as previously described (16).

### scRNAseq

Each sample for sequencing was prepared from triplicate cultures from each individual, which were combined. Each participant was sequenced separately to account for biological differences. Cells submitted for scRNAseq were harvested on ALI day 28–30, after confirming the presence of cilia by direct visualization and immunofluorescent staining using a cilia marker to confirm the presence of well-differentiated multiciliated cells. Cultured cells were briefly washed using prewarmed medium 24 hours before collection. To obtain single cell suspensions, cells were incubated for 5–7 minutes with 100 μL of warm 0.05% trypsin solution and detached from the culture filter by gently scraping the Transwell membrane using a pipet tip. Collected cells were incubated for an additional 5–7 minutes in trypsin and then resuspended in a solution of 0.04% BSA in PBS. Cell viability assay was confirmed to be above 80% for all downstream sequencing analysis.

Library preparation and sequencing was performed by the Genome Technology Access Center at Washington University in St. Louis. A minimum of 20,000 cells per sample were submitted for cell isolation using a chromium Controller (10X Genomics) for single cell capture, and cDNA was prepared according to the 10x Genomics protocols as described in Supplemental Methods. A total of 7,000 to 10,000 cells were sequenced per sample. Paired-end sequencing reads were processed by Cell Ranger (10X Genomics software, version 2.0.0). Reads were aligned to the GRCh38 (version 90) for genome annotation, demultiplexing, barcode filtering, and gene quantification.

### scRNA analysis

To assess the differences in the composition of cell populations, we performed global unsupervised clustering analysis of sequenced cells using the Seurat R package version 4.3 (64). Low-quality cells with less than 2,000 genes were removed from analysis and a cutoff of less-than 25% mitochondrial genes content was chosen. Genes that were detected in less than 3 cells were also filtered out. Doublet detection and removal was performed with DoubletFinder on Seurat objects created from each 10X lane using a presumed doublet rate of 7.5%. The 13 data sets were integrated using the Seurat reciprocal PCA (RPCA) pipeline to reduce the computational burden. Following unsupervised Louvain clustering and UMAP visualization, cell type–specific markers were determined among clusters using Seurat’s *FindAllMarkers* function, with the log fold-change set to 0.25. Annotation for broad cell types (basal, ciliated, secretory, dividing, and ionocytes) was performed using known markers from previously published data sets (65–67), and marker genes were identified for subclusters within these categories through curation of the *FindAllMarkers* gene lists.

### Trajectory analysis

For RNA velocity analysis, spliced and unspliced mRNAs for all genes on a per-cell basis were determined using Velocyto (68) (https://github.com/velocyto-team/velocyto.py; Branch: Master, Commit ID: 0963dd2df0ac802c36404e0f434ba97f07edfe4b) using a cell sorted BAM file for each of control samples as input. Resulting loom files were input to scVelo, merged in an anndata object, and velocities were estimated using *scv.tl.velocity*. For visualization, cluster assignments and UMAP coordinates for each cell were imported to the anndata object metadata from our existing Seurat object. Following conversion of a subsetted Seurat object containing all control cells to a SingleCellExperiment format, further trajectory inference was performed with the R package Slingshot using default parameters, with the Bas2 cluster provided as a start point for the trajectory.

### Differential expression testing, gene ontology, and visualization

To identify variation in expression patterns across our 3 groups of participants, broad cell types (basal, secretory, or ciliated) were organized into subsets from the combined object and identities set to ‘group’ (control, mother, and PCD), then *FindAllMarkers* was run with log-fold change set to 0.25. To identify specific marker genes up or downregulated in PCD and heterozygotes versus controls, gene expression was compared between PCD and control or mother and control using the Seurat function FindMarkers for each major cell type, with significance cutoff of 0.05 (using Bonferroni-corrected adjusted *P* values) and log-fold change set to 0.2. For gene ontology/pathway analysis marker gene lists were input to Enrichr (mayyanlab.cloud/enrichr), with table output arranged by adjusted *P* value and manual curation to remove uninformative or redundant ontologies. For select visualizations we employed the R package plot1cell (https://github.com/TheHumphreysLab/plot1cell; Branch: Master, Commit ID: 8b0310a0eb8ffd5e3bf7ff266380757fcdc61a2d). Where performed, integration with external public data sets was preceded by *SCTransform* v2 normalization of each data set, with *PrepSCTFindMarkers* run on the integrated object to obtain an expression matrix corrected for differences in sequencing depth for use in downstream DEG testing. A generalized mixed model was used to assess transcriptional changes in the data set that included external public data sets (69).

### Bulk RNAseq and analysis

For bulk RNAseq analysis, airway cells were harvested from 3 separate Transwell cultures and sequenced as triplicates. Total RNA was collected using the Total RNA extraction kit (Qiagen). Total RNA integrity was determined using Agilent Bioanalyzer. Library preparation and sequencing was performed by the Genome technology Access Center at Washington University in St. Louis, as described in Supplemental Methods. Differential expression analysis was then performed to analyze for differences between conditions and the results were filtered for only those genes with Benjamini-Hochberg FDR adjusted *P* values less than or equal to 0.05.

### Immunofluorescence

Airway cells were fixed and immunostained as previously described (18). Primary antibodies used are shown in Supplemental Table 7. Primary antibodies were detected using Alexa Fluor-488, Alexa fluor-555, and Alexa Fluor-647 (Life Technologies). The DNA was stained using 4′, 6-diamidino-2-phenylindole (DAPI, Vector Laboratories). Images were acquired using a Nikon Ti2 microscope interfaced to a CMOS camera and Elements imaging software (NIS-elements). Images were globally adjusted for brightness and contrast using Affinity Photo (v.2.5, Serif Ltd).

### Lentiviral production

shRNA sequences were obtained from Sigma-Aldrich in the pKL0.1 vector and were chosen from the Broad Institute RNAi library based on specificity and efficacy and obtained from Sigma-Aldrich in the pKL0.1 vector (Supplemental Table 5). Gene KDs were performed using our previously published protocols (33, 35).

### High-speed video microscopy of multiciliated cells

Cultured cells were imaged live and recorded using a Nikon Eclipse Ti-U inverted microscope. The microscope was enclosed in a customized environmental chamber maintained at 37°C as described (35). Images were captured by a high-speed video CMOS camera and processed with the Sisson-Ammons Video Analysis system (Ammons Engineering) (70). CBF was analyzed in at least 3 fields obtained from triplicate culture of each preparation, in cultured triplicates, after visually confirming ciliated cells in the analyzed areas. We measured ciliary transport by tracking the displacement of microbeads (Fluoresbrite, 2 μm diameter; Polysciences) on the surface of ciliated cells in triplicate cultures, as previously described (71).

### Protein analysis

Cells were lysed in RIPA buffer (Thermo Fisher Scientific). Protein concentration was measured using the Pierce BCA Assay (Thermo Fisher Scientific). Ten micrograms of proteins for each well were separated on 10% SDS-PAGE gels along with prestained molecular weight markers (161-0374, Bio-Rad) and transferred to a polyvinylidene fluoride (PVDF) membrane (Sigma-Aldrich). The PVDF membrane blots were incubated overnight at 4°C with the primary antibodies. Immunoblots were imaged and analyzed using the LICOR Imaging System (LI-COR Biosciences).

### Liquid chromatography and mass spectrometry

Protein extraction and digestion and subsequent peptide analysis were carried out as reported by LaPak et al. (72). Briefly, cells were lysed in a urea lysis buffer containing 8 M urea, 1 mM EDTA, 50 mM tris (pH 8.0), 70 mM NaCl, and protease and phosphatase inhibitors. We used a custom internal standard–triggered PRM (IS-PRM) method to quantify peptides on an Orbitrap Eclipse Tribrid mass spectrometer (Thermo Fisher Scientific). Each sample was run in technical triplicate. Logarithmically transformed protein abundances and fold changes are reported in Supplemental Table 4.

### Electron microscopy

For immunolocalization of protein at the ultrastructural level using immunogold labeling, cells were fixed in 4% paraformaldehyde and 0.05% glutaraldehyde (Polysciences Inc.) in 100 mM PIPES and 0.5 mM MgCl_2_, pH 7.2 buffer for 1 hour at 4°C, and processed as described in Supplemental Methods. All labeling experiments were conducted in parallel with controls omitting the primary antibody.

For SEM preparations, cultured airway epithelial cells were treated with PHEM buffer containing 60 mM PIPES, 25 mM HEPES, 10 mM EGTA, 2 mM MgCl_2_, pH 6.9 with 1% Triton X100] buffer for 1 minute at room temperature to remove the ciliary membrane, and processed as described in Supplemental Methods.

### Chlamydomonas studies

#### Strains.

*Chlamydomonas* strains CC-124 (WT), CC-2228 (*oda1* mutant), CC-3007 (*oda3* mutant), CC-4533 (WT), and LMJ.RY0402.183043 (Cre16.g682725, gst2 mutant) were used for these studies. The insertional mutants were obtained from the *Chlamydomonas* library project (CLiP) collection. The mutant stains were first backcrossed to show that the insertion cosegregates with the drug resistance cassette in 12 tetrads. A second cross is made with 2 strains that lack the a “clumping” phenotype, which is often seen in mutants and 24 tetrads of each cross were examined. The drug resistance and the swimming phenotype cosegreate in these 48 tetrads. A third backcross was performed and progeny for this backcross were used for downstream experiments. Cells were plated onto R (rich) medium plates for 2 days at 25°C (73, 74), then transferred into 1 mL of liquid R medium in a 1.5 mL Eppendorf tube and rocked under light for 3 hours to allow cilia to assemble. Strain LMJ.RY0402.127518 was mated to CC-124, and 20 meiotic progeny were obtained. The insertion of *aphviii* as ascertained by PCR and resistance to paramomycin cosegregated together with the slowed swimming as examined by behavior with phase microscopy at × 40.

### Axoneme isolation and mass spectrometry

Axonemes of WT strain CC-124 and mutant strains CC-2228 and CC-3007 were prepared as sets of 3 biological replicates using the dibucaine method (75). From each sample, 50 μg of protein from each sample was submitted for tandem mass spectrometry analysis to the Danforth Plant Science Center St. Louis, Missouri for analysis, as described previously (38).

### Chlamydomonas swimming velocity and CBF analysis

*Chlamydomonas* cells were grown as previously described (76). Bright field microscopy was carried out in a climate-control room maintained at 21°C. Recordings were performed using 10 μL from liquid cell cultures (after inducing gametogenesis by removal of nitrogen) using a Zeiss Axiophot (Carl Zeiss AG) with a × 40 Plan-Neofluar objective lens as previously described (38). Videos were analyzed using ImageJ (Fiji) (77) to create a binary file only displaying the cells. Cells were tracked using the 2D/3D single-particle tracking tool of the MosaicSuite for Fiji (MOSAIC Group, MPI-CBG). A custom-made program written in Matlab R2016a (The Mathworks) was then used to compute the cells velocity, as well as the CBF extracted by Fast-Fourier-Transform of the trajectory.

### Statistics

Analysis was performed using GraphPad Prism (version 10). Differences between 2 groups were compared using the Mann-Whitney *U* test. Multiple medians were compared using the Kruskal-Wallis test, followed by Dunn’s correction for multiple comparisons. Paired comparisons were analyzed using the Wilcoxon signed-rank test. Differential gene expression comparisons were made using nonparametric Wilcoxon rank sum test. PCR comparison between sequential time points was performed using a *t* test with Welsh’s correction. *P* < 0.05 indicated a statistically significant difference.

### Study approval

The present studies using human nasal epithelial cells were reviewed and approved by the IRB of Washington University in St. Louis. Written informed consent was obtained from individuals (or their legal guardians). All human samples were deidentified. Human tracheal cells were isolated from surgical excess of tracheobronchial segments of nondiseased lungs donated for transplantation. These unidentified tissues are exempt from regulation by HHS regulation 45 CFR Part 46.

### Data availability

Sequencing data are available at the Gene Expression Omnibus, under accession no. GSE272189. Other data are available in the Supporting Data Values file and upon request.

## Author contributions

JRK, DKG, and NH performed bioinformatic analysis. DKG, WKT, HX, GMP, MLB, NTW, and AB performed experiments. FJH, MBM, SKD, SLB, and AH provided reagents. SKD designed and supervised *Chlamydomonas reinhardtii* studies. MBM supervised mass spectrometry studies. FJH supervised the iPSc studies. AH and SLB conceived the work, designed experiments, analyzed results, and wrote the manuscript with JRK. SKD and MBM reviewed and edited the manuscript.

## Supplementary Material

Supplemental data

Unedited blot and gel images

Supplemental table 1

Supplemental table 2

Supplemental table 3

Supplemental table 4

Supplemental table 6

Supplemental video 1

Supplemental video 2

Supplemental video 3

Supporting data values

## Figures and Tables

**Table 1:**
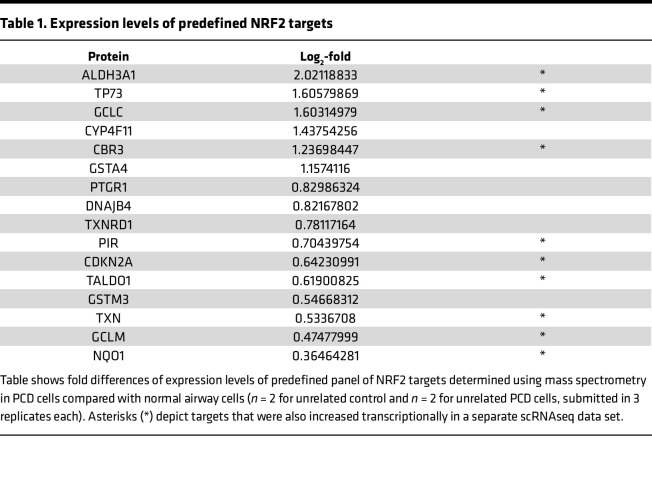
Expression levels of predefined NRF2 targets

## Version 1 07/23/2024

In-Press Preview

## Version 2 09/10/2024

Electronic publication
