# Supplementary material for: Transcriptional analysis of primary ciliary dyskinesia airway cells reveals a dedicated cilia glutathione pathway
Source: JCI Insight. 2024 Jul 23;9(17):e180198. doi: 10.1172/jci.insight.180198 (PMC11385084; doi:10.1172/jci.insight.180198)
Supplement: Supplemental data [file jciinsight-9-180198-s199.pdf]

Supplemental Material

Transcriptional analysis of primary ciliary dyskinesia airway cells reveals a dedicated cilia glutathione pathway

Jeffrey R Koenitzer[1], Deepesh Kumar Gupta[2], Wang Kyaw Twan[2], Huihui Xu[2], Nicholas Hadas[2], Finn J Hawkins[3,4], Mary Lou Beermann[3], Gervette M Penny[5], Nathan T Wamsley[6], Andrew Berical[3,4], M Ben Major[6], Susan K Dutcher[5,6], Steven L Brody[1], Amjad Horani[2,6]

[1] Department of Medicine, Washington University School of Medicine, St. Louis, Missouri, USA

[2] Department of Pediatrics, Washington University School of Medicine, St. Louis, Missouri, USA

[3] Center for Regenerative Medicine of Boston University and Boston Medical Center, Boston, MA, USA

[4] The Pulmonary Center and Department of Medicine, Boston University and Boston Medical Center, Boston, MA, USA

[5] Department of Genetics, Washington University School of Medicine, St. Louis, Missouri, USA

[6] Department of Cell Biology and Physiology, Washington University School of Medicine, St. Louis, Missouri, USA

Corresponding author:

Amjad Horani, MD

Department of Pediatrics, 660 South Euclid Avenue, Mailbox 8116. St. Louis, Missouri, 63110.

Telephone: 314 286 2886, Facsimile: 314 454 2515. e-mail: horani_a@wustl.edu

ORCID: 0000-0002-5352-1948

The authors have declared that no conflict of interest exists.

Supplement contains:

Supplemental Methods.

Supplemental Tables 1, 5, 7.

Supplemental Figures 1-6.

Supplemental methods

Single-cell RNA sequencing

Cultured primary airway cells were prepared for scRNAseq by dissociating the ALI cultures. Cell viability was maintained above 80% across all samples. Library preparation and sequencing was performed by the Genome Technology Access Center at Washington University in St. Louis. For each sample, 20,000 cells were loaded on a Chromium Controller (10x Genomics) for single cell capture and cDNA was prepared according to the 10x Genomics protocols. cDNA was prepared after Gel Bead-in Emulsion (GEM) generation and barcoding, followed by the GEM-RT reaction and bead cleanup steps using the manufacturer protocols. Purified cDNA was amplified for 11 to 13 cycles before cleanup using SPRI select beads. Samples were then run on an Agilent Bioanalyzer to determine cDNA concentration. Gene expression (GEX) libraries were prepared using the 10X Genomics Chromium Single Cell 3' Reagent Kits as recommended by the User Guide (v3.1 Chemistry Dual Index) with appropriate modifications to the PCR cycles based on the calculated cDNA concentration. For sample preparation on the 10X Genomics platform, the Chromium Next GEM Single Cell 3' Kit v3.1(16 reactions, PN-1000268), Chromium Next GEM Chip G Single Cell Kit (48 reactions, PN-1000120), and Dual Index Kit TT Set A (96 reactions, PN-1000215) were used. The concentration of each library was accurately determined through qPCR utilizing the KAPA library Quantification Kit according to the manufacturer's protocol (KAPA Biosystems/Roche) to produce cluster counts appropriate for the Illumina NovaSeq6000 instrument. Normalized libraries were sequenced on a NovaSeq6000 S4 Flow Cell using the XP workflow and a 50x10x16x150 sequencing recipe according to manufacturer's protocol. A median sequencing depth of 50,000 reads/cell was targeted for each Gene Expression Library. Paired-end sequencing reads were processed by Cell Ranger (10X Genomics software, version 2.0.0). Reads were aligned to the GRCh38 (version 90) for genome annotation, demultiplexing, barcode filtering, and gene quantification. To account for empty droplets,

barcodes that had less than 10% of the 99th percentile of total unique molecular identifiers (UMI) counts per barcode were removed from analysis. Gene barcode matrices for each sample were generated by counting the number of UMIs for a given gene (as a row) in the individual cell (as a column).

Bulk RNA Sequencing and Analysis

Total RNA was collected using the Total RNA extraction kit (Qiagen, Germany). Total RNA integrity was determined using Agilent Bioanalyzer. Library preparation and sequencing was performed by the Genome Technology Access Center at Washington University in St. Louis, as described in Supplemental Methods. Library preparation was performed with 10ng of total RNA with a Bioanalyzer RIN score greater than 8.0. ds-cDNA was prepared using the SMARTer Ultra Low RNA kit for Illumina Sequencing (Takara-Clontech) per the manufacturer's protocol. cDNA was fragmented using a Covaris E220 sonicator set at a peak incident power 18, duty factor 20%, and cycles per burst 50 for 120 seconds. cDNA was blunt ended, modified by addition of an A base to the 3' ends, followed by ligation of Illumina sequencing adapters ligated to the ends. Ligated fragments were amplified for 12-15 cycles using primers incorporating unique dual index tags. Fragments were sequenced on an Illumina NovaSeq-6000 using paired-end reads extending 150 bases. Base calls and demultiplexing were performed with Illumina's bcl2fastq software. RNA-seq reads were then aligned to the Ensembl release 76 primary assembly with STAR version 2.5.1a (1). Gene counts were derived from the number of uniquely aligned unambiguous reads by Subread:featureCount version 1.4.6-p5 (2). Isoform expression of known Ensembl transcripts were estimated with Salmon version 0.8.2 (3). Sequencing performance was assessed for the total number of aligned reads, total number of uniquely aligned reads, and

features detected. The ribosomal fraction, known junction saturation, and read distribution over known gene models were quantified with RSeQC version 2.6.2.

All gene counts were then imported into the R/Bioconductor package EdgeR and TMM normalization size factors were calculated to adjust for samples for differences in library size. Ribosomal genes and genes not expressed in the smallest group size minus one sample greater than one count-per-million were excluded from further analysis. The TMM size factors and the matrix of counts were then imported into the R/Bioconductor package Limma. Weighted likelihoods based on the observed mean-variance relationship of every gene and sample were then calculated for all samples and the count matrix was transformed to moderated log 2 counts-per-million with Limma's voomWithQualityWeights. The performance of all genes was assessed with plots of the residual standard deviation of every gene to their average log-count with a robustly fitted trend line of the residuals. Differential expression analysis was then performed to analyze for differences between conditions and the results were filtered for only those genes with Benjamini-Hochberg false-discovery rate adjusted p-values less than or equal to 0.05.

Electron Microscopy

For immunolocalization of protein at the ultrastructural level using immunogold labeling, cells were fixed in 4% paraformaldehyde and 0.05% glutaraldehyde (Polysciences Inc., Warrington, PA) in 100mM PIPES and 0.5mM $MgCl_2$, pH 7.2 buffer for 1 hour at 4°C. Samples were then embedded in 10% gelatin and infiltrated overnight with PIPES/$MgCl_2$ buffer containing 2.3M sucrose and 20% polyvinyl pyrrolidone at 4°C. Samples were trimmed, frozen in liquid nitrogen, and sectioned with a Leica Ultracut UCT7 cryo-ultramicrotome (Leica Microsystems Inc., Bannockburn, IL). Ultrathin sections of 50nm were blocked with PIPES/$MgCl_2$ buffer containing 5% fetal bovine serum (FBS) and 5% normal goat serum (NGS) for 30 min and subsequently incubated with rabbit

anti-GSTA2 antibody (Proteintech) for 1 hour at room temperature. Following washes in blocking buffer, sections were incubated with goat anti-rabbit IgG (H+L) conjugated to 12 nm colloidal gold (Jackson ImmunoResearch Laboratories, Inc., West Grove, PA) for 1 hour. Sections were stained with a buffer containing 0.3% uranyl acetate and 2% methyl cellulose and viewed on a JEOL 1200 EX transmission electron microscope (JEOL USA Inc., Peabody, MA) equipped with an AMT 8-megapixel digital camera and AMT Image Capture Engine V602 software (Advanced Microscopy Techniques, Woburn, MA). All labeling experiments were conducted in parallel with controls omitting the primary antibody.

For scanning electron microscopy (SEM) preparations, cultured airway epithelial cells were treated with PHEM buffer containing 60 mM PIPES, 25 mM HEPES, 10 mM EGTA, 2 mM $MgCl_2$, pH 6.9 with 1% Triton X100] buffer for 1 minute at room temperature to remove the ciliary membrane. Samples were then fixed overnight in a solution containing 4% paraformaldehyde in PBS, pH 7.4 at 4 °C. Cells were then rinsed in PBS three times and transferred into a solutions of 50 mM glycine in PBS for 15 minutes to quench aldehydes. Following this, samples were first incubated in 1% BSA in PBS for 30 minutes and then labelled with the GSTA2 antibody overnight at 4 °C. After washing the samples 5 times for 5 minutes each in 1% BSA in PBS, samples were incubated with anti-rabbit (Jackson Immuno Research, 711-205-152) secondary antibody prepared at 1:20 dilution for 1 hour. Samples were then washed 5 times for 5 minutes each in PBS, treated with 2% glutaraldehyde in PBS for 15 minutes and washed again in PBS. Following this, cells on membranes were incubated with 0.5% osmium tetroxide for 20 minutes on ice and then washed in Ultrapure water 3 times for 10 minutes each. Samples were then dehydrated in a graded ethanol series (10%, 20%, 30%, 50%, 70%, 90%, followed by 3 times in 100%) for 5 minutes in each step and loaded into a critical point drier (Leica EM CPD 300, Vienna, Austria) that was set to perform 12 CO_2 exchanges at the slowest speed. Cells on membranes were then mounted on aluminum stubs with carbon adhesive tabs and coated with 12 nm of carbon (Leica

ACE 600, Vienna, Austria). SEM images were acquired on a FIB-SEM platform (Helios 5 UX DualBeam Fisher Scientific, Brno, Czech Republic) using SEM imaging mode at 5 kV and 0.1 nA. Thermoluminescent dosimeter detector (TLD) was used for acquisition of secondary electron signal and In-Column detector (ICD) was used for acquisition of backscattered electron signal. Additional images representing a combination of TLD and ICD signals were acquired using digital image enhancement/mixing module.

Supplemental Tables

Supplemental Table 1. Clinical characteristics of donors

Supplemental Table 2. scRNAseq differentially expressed genes.

Supplemental Table 3. Bulk RNAseq analysis comparing PCD variant cells to control healthy cells.

Supplemental Table 4. Mass spectrometry fold changes of predefined NRF2 targets shown in Table 1.

Supplemental Table 5. shRNA sequences.

Supplemental Table 6. Protein levels of isolated mutant *Chlamydomonas* cilia.

Supplemental Table 7. Antibodies used.

Supplemental Table 1. Clinical characteristics of donors

	Gender	Age (years)	Sinopulmonary disease	Genetic	TEM	nNO
**Control 1 (hnec102)**	Female	30s	None	No path variants		
**Control 2 (hnec103)**	Male	40s	None	No path variants		
**Control 3 (hnec104)**	Male	60s	None	No path variants		
**Control 4 (hnec105)**	Male	60	None	No path variants		
**Control 5 (hnec106)**	Male	20s	None	No path variants		
**Patient 1 (PCD111)**	Female	16	Yes	c.12617G>A<br>c.12617G>A	ODA defect	14.2 nl/min
**Patient 2 (PCD117)**	Male	18	yes	c.13458dupT	ODA defect	7.3 nl/min
**Patient 3 (PCD129)**	Female	6	Yes	c.5665_5666delCT<br>c.5992 G>A	ODA defect	7 nl/min
**Patient 4 (PCD138)**	Female	4	Yes	c.12500-14 A>G<br>c.11758_11668del	ODA defect	3nl/min
**Parent 1 (PCD111M)**	Female	40s	no	Heterozygous		
**Parent 2 (PCD 117M)**	Female	40s	no	Heterozygous		
**Parent 3 (PCD129M)**	Female	40s	No	Heterozygous		
**Parent 4 (PCD138M)**	Female	20s	No	Heterozygous		

Supplemental Table 5 – shRNA sequences

shRNA	TRC Clone ID	Sequence
1	TRCN0000152045	CCCATGGATGAGAAATCTTTA
2	TRCN0000423433	CCTATTCACCCACTTAGTTAA
3	TRCN0000155838	CTCAACTACATTGCCAGCAAA

Supplemental Table 7 – Antibodies used

**Antibody**	**Species**	**Concentration**	**Cat #**	**company**
acetylated α-tubulin	Mouse	1:5000 (IF)	clone 6-11-B1	Sigma-Aldrich
GSTA2	Rabbit	1:400 (IF)	10505-1-AP	Proteintech
GSTA2	Mouse	1:1000 (WB)	H00002939-M05	Abnova
GSTA1	Rabbit	1:1000 (IF),<br>1:500 (WB)	HPA053817	Sigma-Aldrich

Supplemental Figures

Supplemental Figure 1

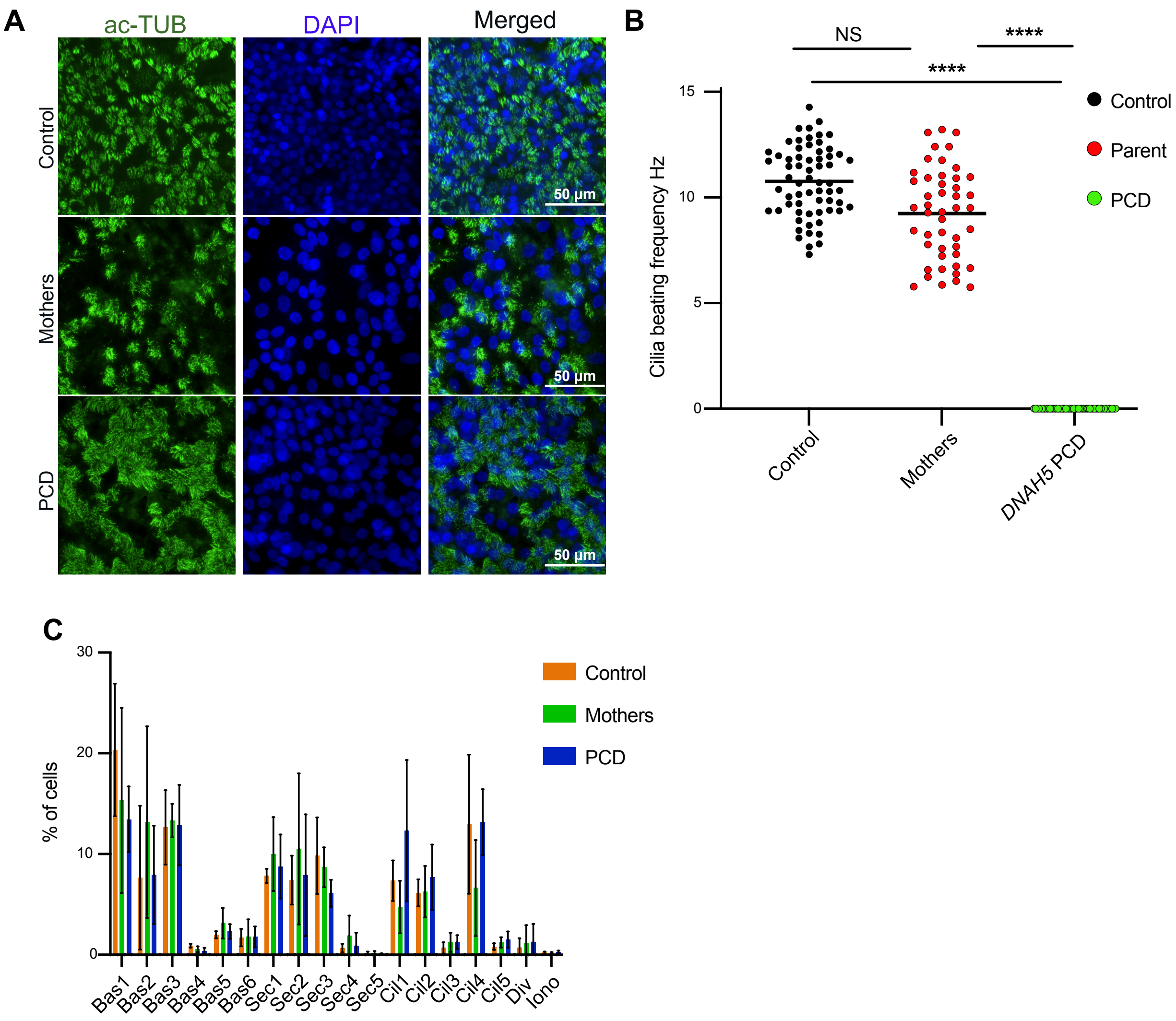

Supplemental Figure 1. Cultured primary nasal airway cells. (A) Representative immunofluorescent staining of cultured cells demonstrating the extent of multiciliated cells and level of ciliation in samples submitted for single cell RNAseq (scRNAseq) analysis. **(B)** Cilia beat frequency (CBF) of cells from PCD, maternal, and normal control groups that were submitted for scRNAseq analysis (CBF mean ± SEM frequency of 10.75±0.21, 9.23±0.32, 0.0±0.0, respectively). CBF was assessed in 3 random areas per Transwell culture; 3 cultures replicated

were performed for each sample. (**C**) Percentage of cell cluster in scRNAseq datasets in each analyzed group. In panels A, B, and C, n=5 controls, 4 heterozygous, and 4 PCD; ****P < 0.0001 determined using Kruskal-Wallis test with Dunn's multiple-comparison test.

Supplemental Figure 2

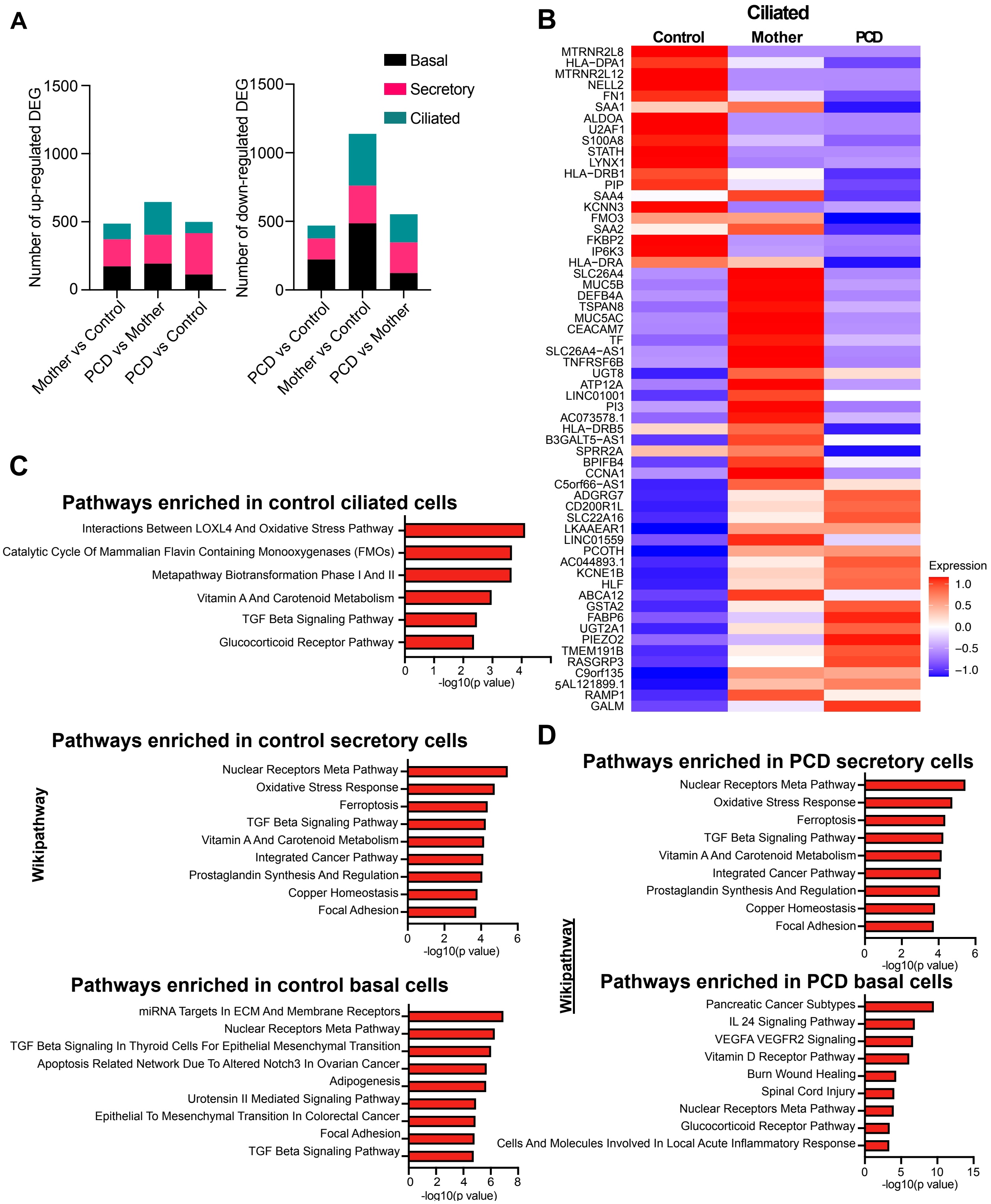

Supplemental Figure 2. Pathway analysis of PCD airway cells. (A) Number of differentially expressed genes observed when comparing primary airway cells from PCD patients to heterozygous maternal cells, and normal healthy cells, distributed by cell type. (**B**) Sub-analysis comparing all female samples across PCD cells, maternal heterozygous cells, and normal control cells. Control cells were supplemented with three additional normal female samples from publicly available datasets (n=4 control, n=4 Mothers, and n=3 PCD). **(C-D)** Pathway analysis of differentially expressed genes in secretory and basal cells of PCD airway cells compared to normal control healthy cells.

Supplemental Figure 3

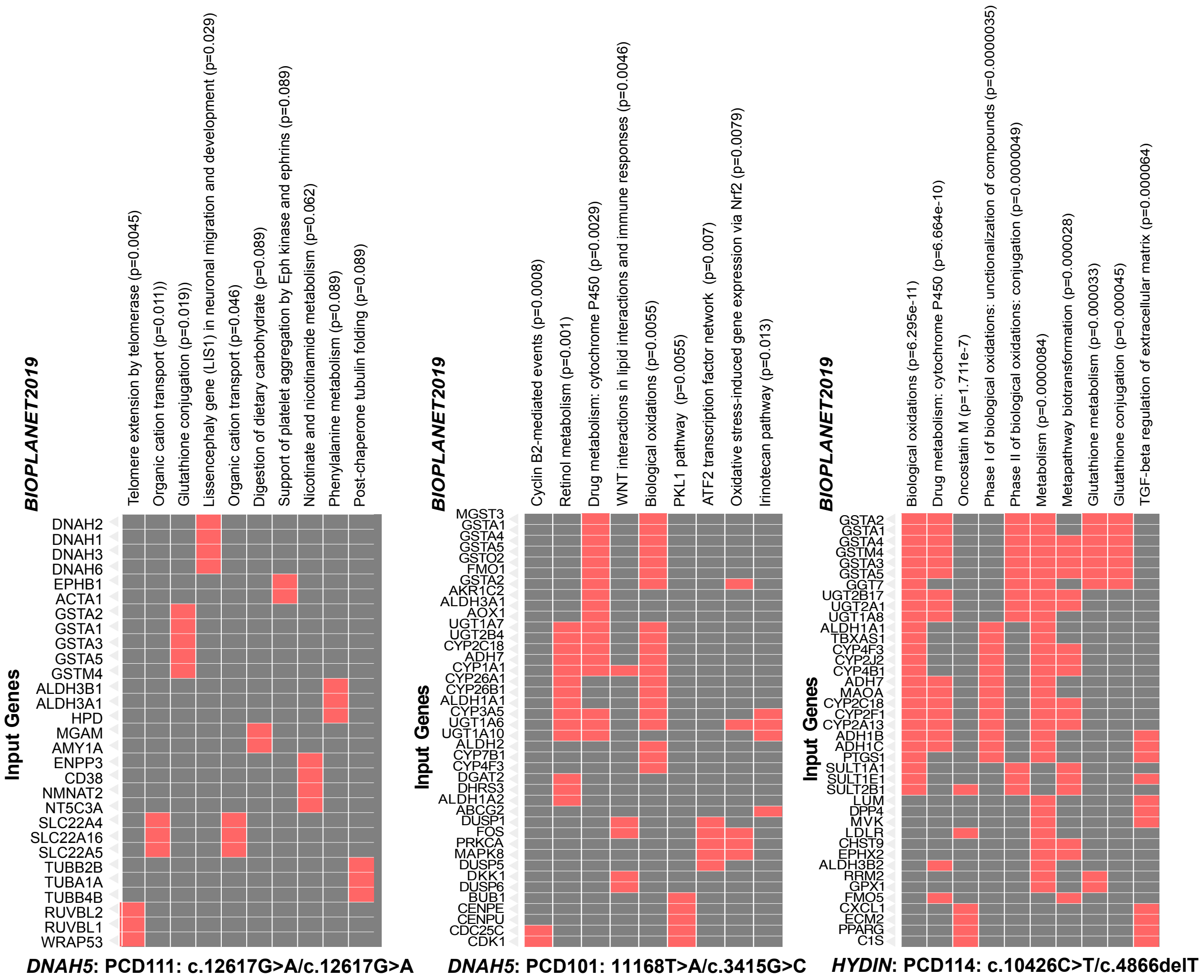

Supplemental Figure 3. Bulk RNAseq analysis of PCD airway cells. Primary nasal cells from three PCD subjects with variants in *DNAH5* and *HYDIN* were included. Clustergram showing top 10 enriched terms, along with their corresponding P values, and top input genes associated with these terms. (n=3 replicates per subject compared to n=2-5 control nasal cells in triplicates).

Supplemental Figure 4

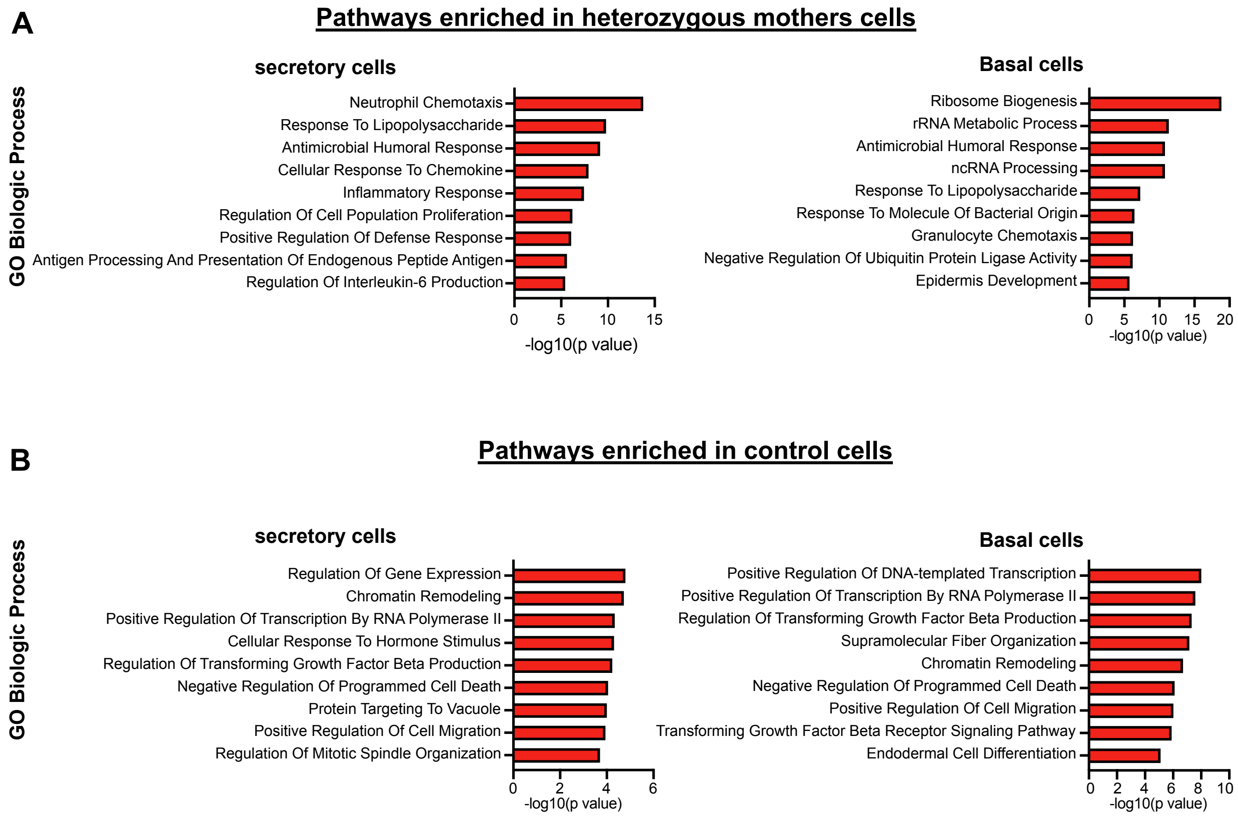

Supplemental Figure 4. Pathway analysis of heterozygous *DNAH5* airway cells. (A-B) Pathway analysis of differentially expressed genes in secretory and basal cells of heterozygous *DNAH5* airway cells compared to control healthy cells.

Supplemental Figure 5

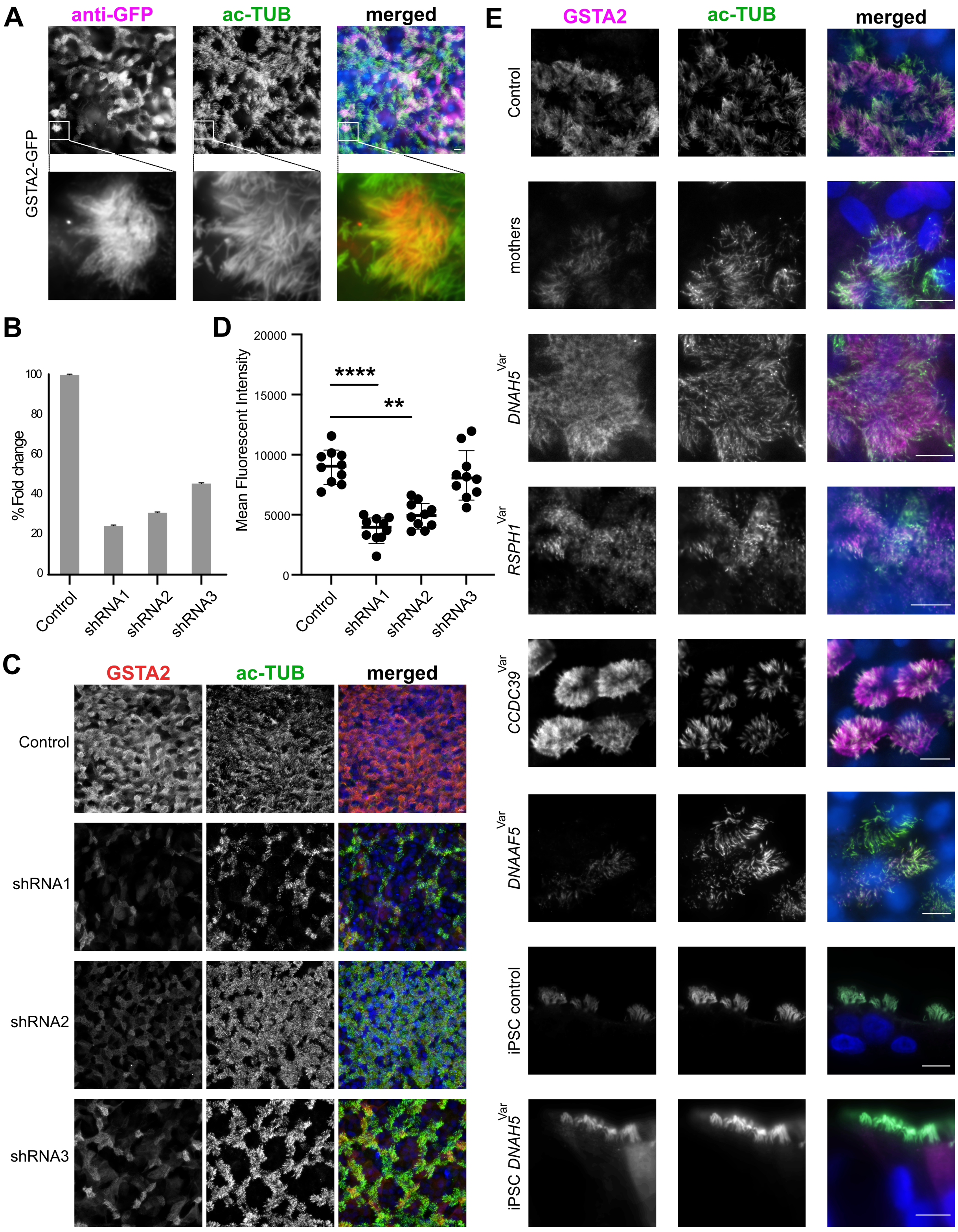

Supplemental Figure 5. *GSTA2* expression in airway cells. (A) Immunofluorescent staining of normal airway cells expressing a C-terminal GFP tagged GSTA2. GSTA2 detection was performed using an antibody against GFP, cilia are detected using an antibody against acetylated-α-tubulin (ac-TUB). Inset shows a closeup of a ciliated cell. (**B**) *GSTA2* expression levels compared using different shRNA sequences (n=3 replicates) detected by RT-PCR. **(C)** Representative images of GSTA2 immunofluorescent staining of different GSTA2 shRNA sequences as in C. **(D)** Mean fluorescent intensity of endogenous GSTA2 expression in multiciliated cells after *GSTA2* knockdown (n=3 replicates per sequence). **(E)** Representative images of GSTA2 immunofluorescent staining of indicated PCD patient samples with indicated gene variant and corresponding to the quantification shown in Figure 7K and 7L. All images were photographed using identical exposures.

Supplemental Figure 6

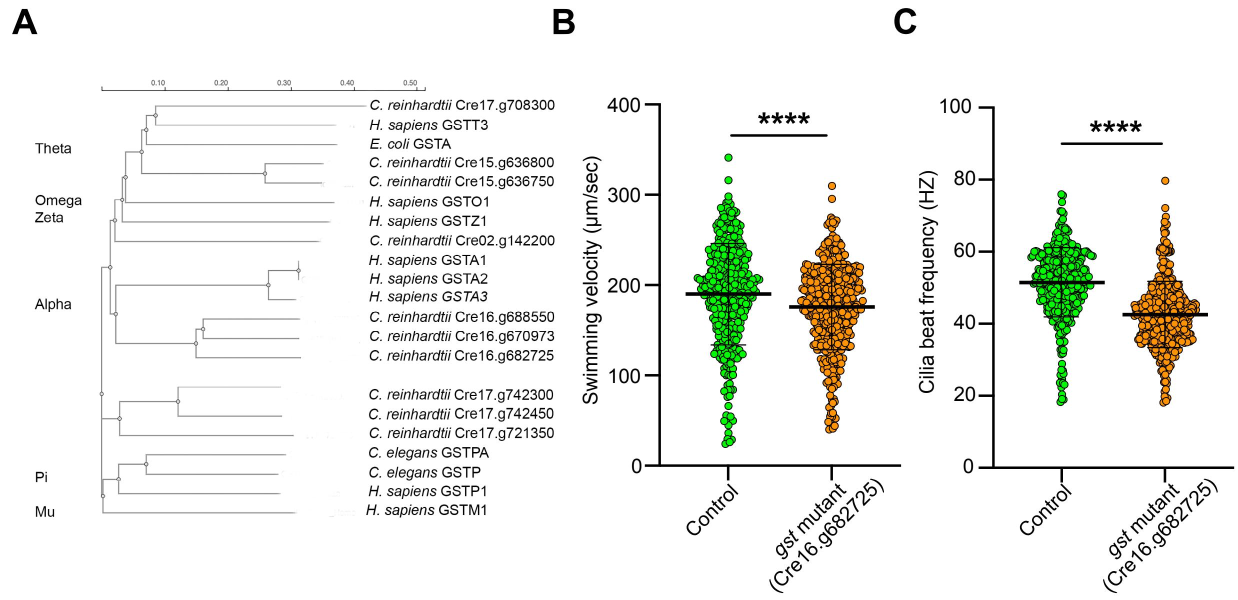

Supplemental Figure 6. GSTA role in ciliated model organism *C. reinhardtii.* (**A**) Phylogenetic tree of glutathione S-transferases. A member of each of the six GST families in humans is included as well as proteins from *E. coli*, *C. elegans* and *C. reinhardtii.* The protein sequences were obtained from Uniprot (https://www.uniprot.org/) or Phytozome (https://phytozome-next.jgi.doe.gov/info/CreinhardtiiCC_4532_v6_1). The tree was assembled using Clustal Omega to achieve a phylogram (4). The proteins encoded by Cre16.g688550, Cre16.g682725, and Cre16.g670973 are orthologs of the human GSTA, GSTA2, and GSTA3 proteins, respectively. *E. coli*: GSTA (P0A9D2). Homo sapiens: GSTT2, Theta family (P0CG30); GSTO1, Omega family (P78417); GSTZ1, Zeta family (G3VB9); GSTA1, Alpha family (P08263); GSTA2, Alpha family (P09210); GSTA3, Alpha family (Q16772); GSTM1, Mu family (P09488); GSTP1, Pi family (P09211). *Caenorhabditis elegans*: GSTP1 (P10299); GSTPA (Q9N4X8). *Chlamydomonas reinhardtii*: Cre17.g742450, Cre17.g742300, Cre17.g721350, Cre16.g688550, Cre16.g682725, Cre16.g670973, Cre15.g636800, Cre15.g636750, Cre02.g142200. *Caenorhabditis elegans*: GSTPA (Q9N4X8); GSTP1 (P10299). (**B**) Swimming velocity analysis of gametic wild-type *Chlamydomonas* strain CC-4533 and Cre16.g682725 named *gst* mutant. (**C**)

Beat frequency analysis of strains in (B). The solid middle line represents the mean of each dataset. Cells cultured from 3 and 4 individual colonies were used to collect motility measurements for CC-4533 and *gst mutant* respectively. Swimming velocity analysis was performed according to methods described in (5). In panel B and C, ****<0.0001 respectively. Line represents the mean. Error bars represent standard deviation.

Supplemental Videos

Supplemental Video 1. 3D rendering of human airway epithelial cells immunostained with an antibody against GSTA2 (green) and imaged using Spatial Array Confocal microscopy (Nikon AX with NSPARC).

Supplemental Video 2. Normal airway cells transduced with a lentivirus expressing GFP.

Supplemental Video 3. Normal airway cells transduced with a lentivirus expressing GFP-GSTA2.

References

1. Dobin A, Davis CA, Schlesinger F, Drenkow J, Zaleski C, Jha S, et al. STAR: ultrafast universal RNA-seq aligner. *Bioinformatics.* 2013;29(1):15-21.
2. Liao Y, Smyth GK, and Shi W. featureCounts: an efficient general purpose program for assigning sequence reads to genomic features. *Bioinformatics.* 2014;30(7):923-30.
3. Patro R, Duggal G, Love MI, Irizarry RA, and Kingsford C. Salmon provides fast and bias-aware quantification of transcript expression. *Nat Methods.* 2017;14(4):417-9.
4. Madeira F, Pearce M, Tivey ARN, Basutkar P, Lee J, Edbali O, et al. Search and sequence analysis tools services from EMBL-EBI in 2022. *Nucleic Acids Res.* 2022;50(W1):W276-W9.
5. Bustamante-Marin XM, Horani A, Stoyanova M, Charng WL, Bottier M, Sears PR, et al. Mutation of CFAP57, a protein required for the asymmetric targeting of a subset of inner dynein arms in Chlamydomonas, causes primary ciliary dyskinesia. *PLoS Genet.* 2020;16(8):e1008691.
