# Supplementary material for: Transcriptional analysis of primary ciliary dyskinesia airway cells reveals a dedicated cilia glutathione pathway
Source: JCI Insight. 2024 Jul 23;9(17):e180198. doi: 10.1172/jci.insight.180198 (PMC11385084; doi:10.1172/jci.insight.180198)

Full unedited gel for Figure 6C

NRF2 lane

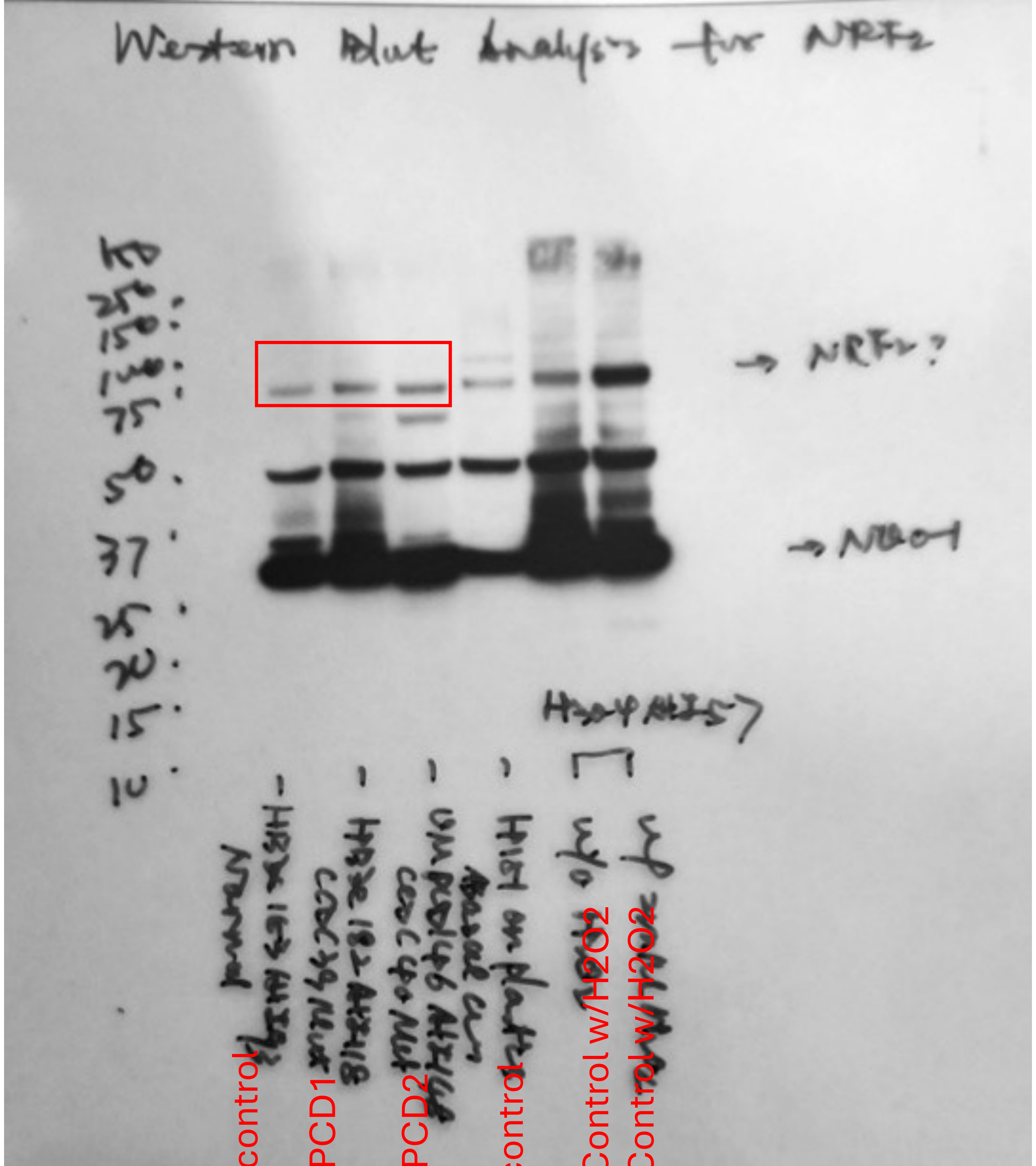

Full unedited gel for Figure 6C

KEPA1 lane

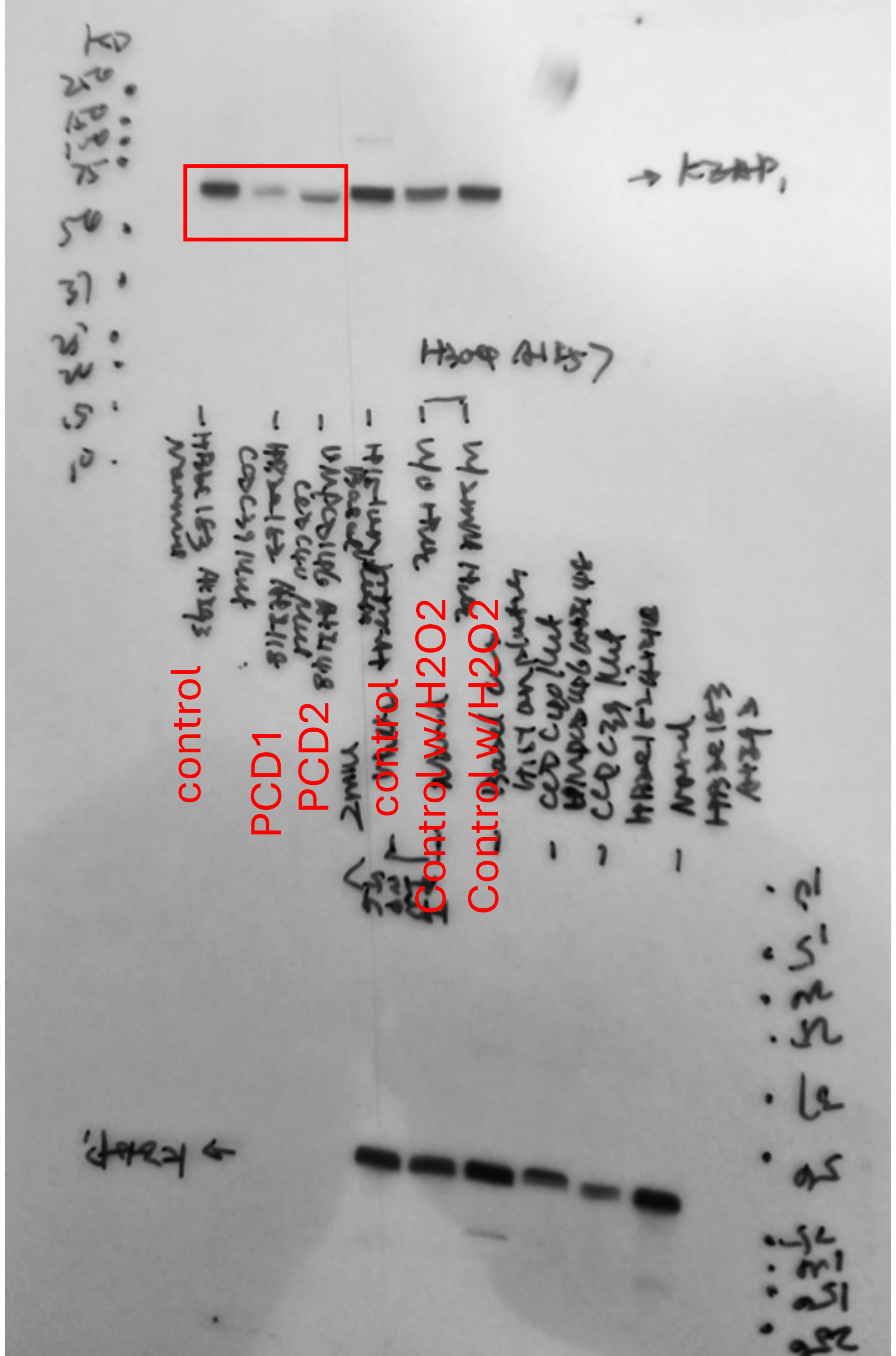

Full unedited gel for Figure 6C

XCT1 lane

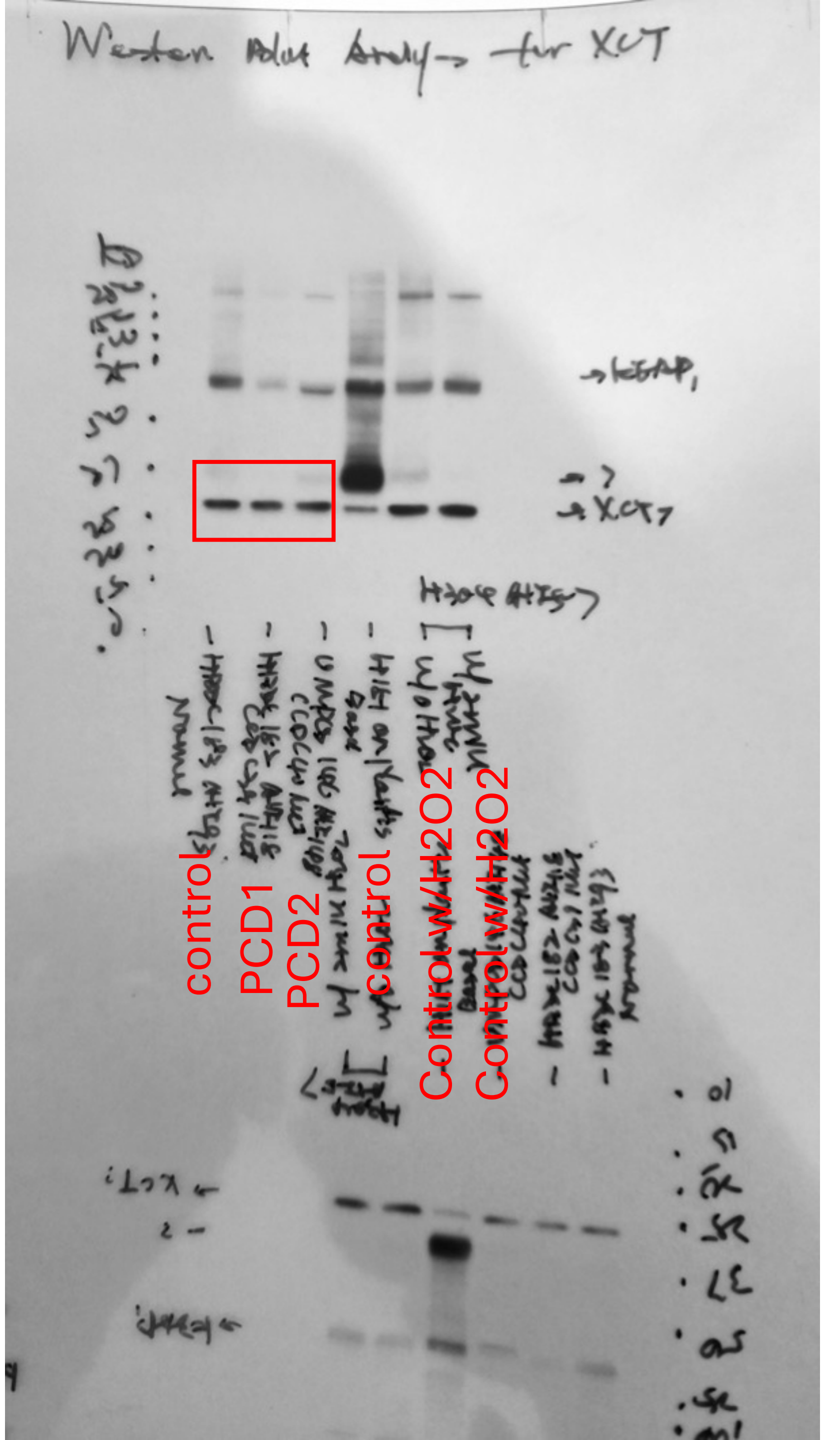

Full unedited gel for Figure 6C

NQO1 lane

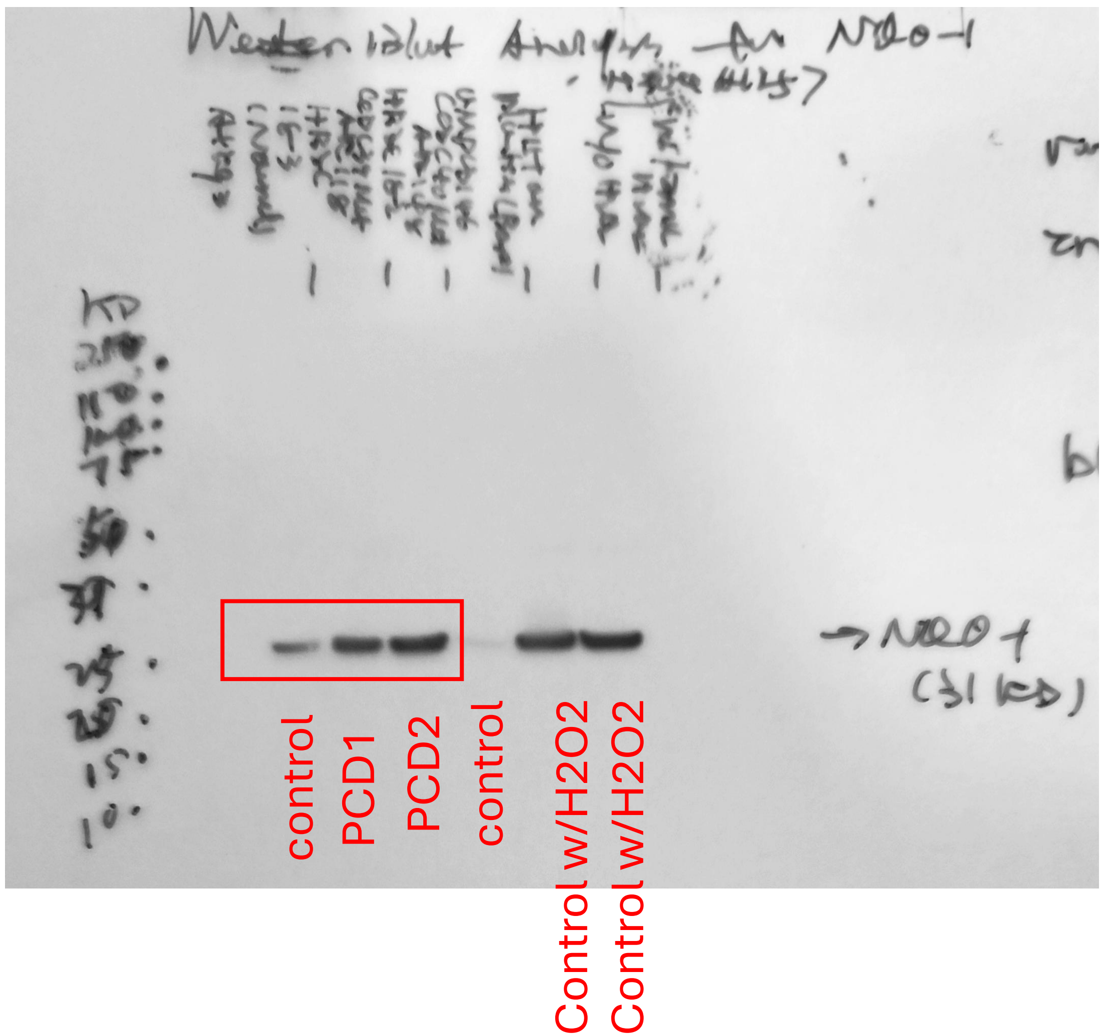

Full unedited gel for Figure 6C

Actin lane

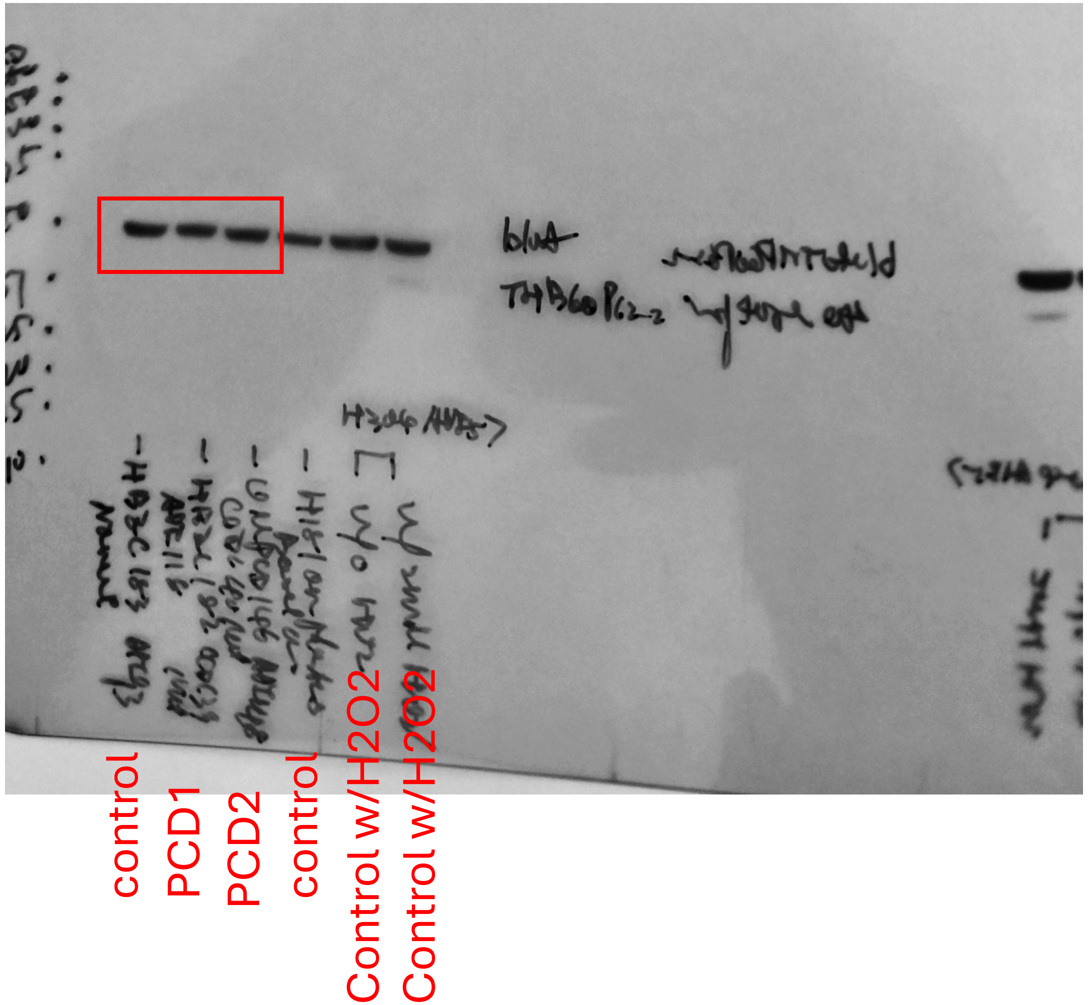

Full unedited gel for Figure 6C

Ac-TUB lane

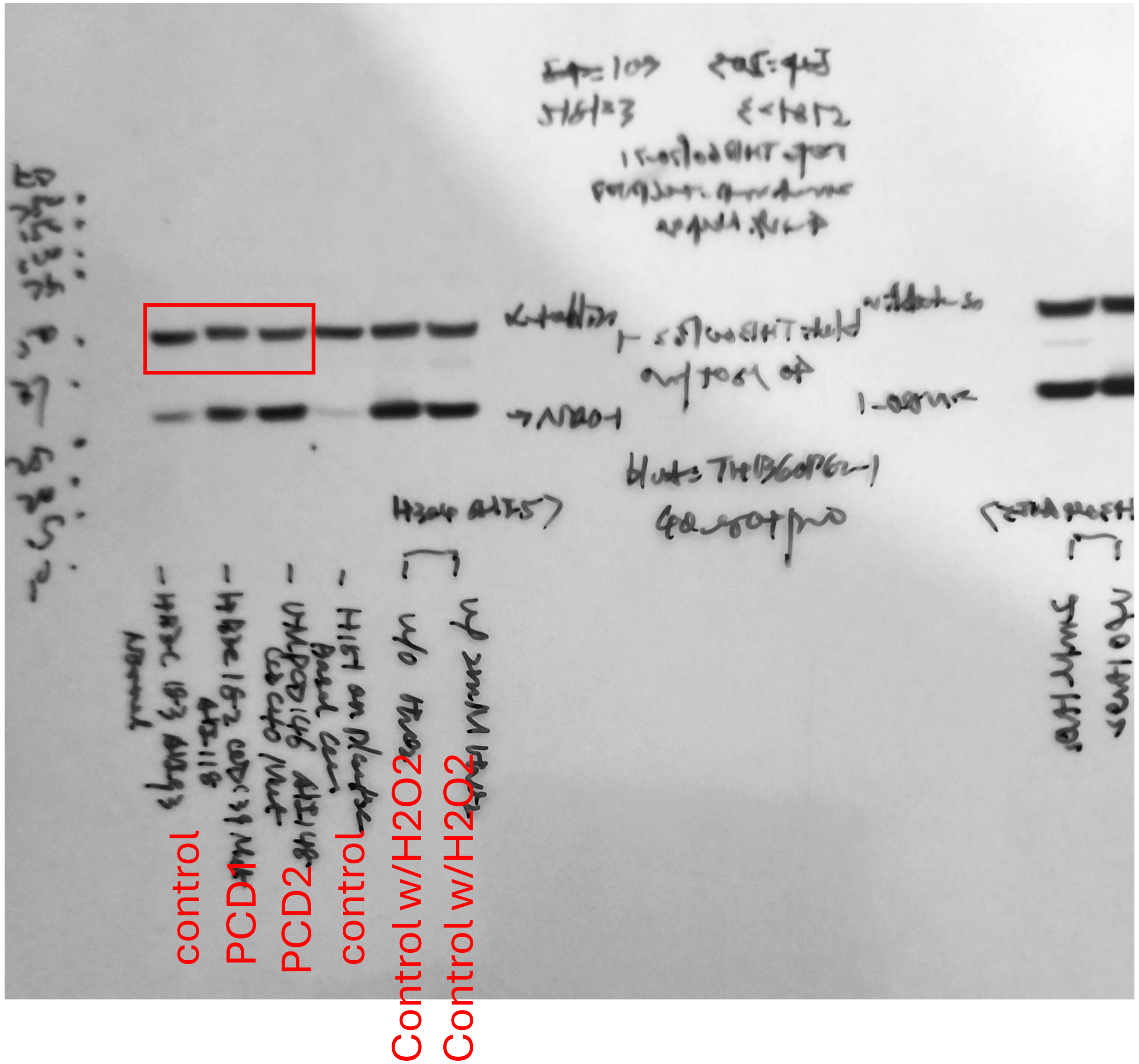

Full unedited gel for Figure 7E

GSTA2 lane

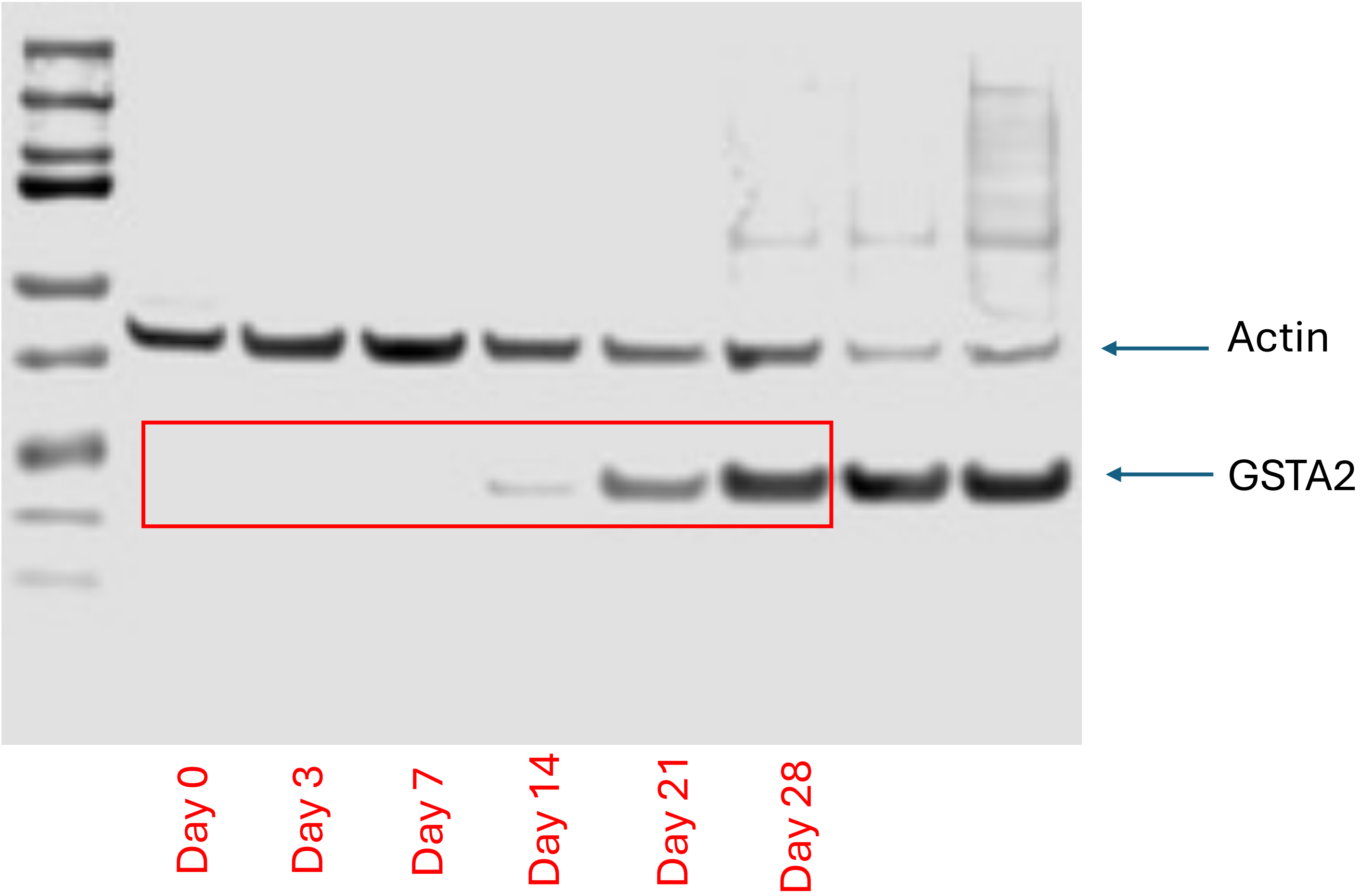

Full unedited gel for Figure 7E

GSTA1 lane

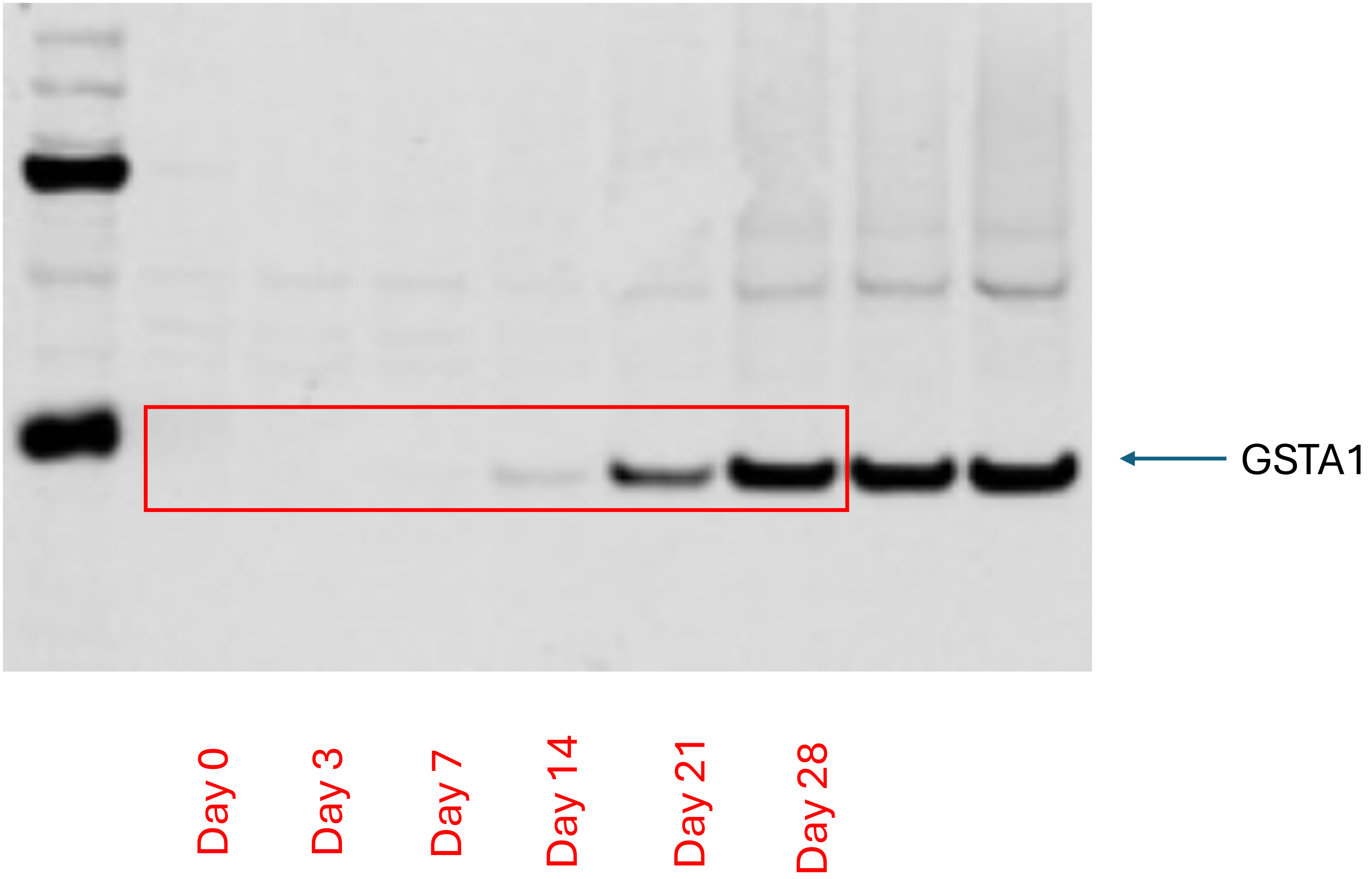

Full unedited gel for Figure 7E

ac-TUB lane

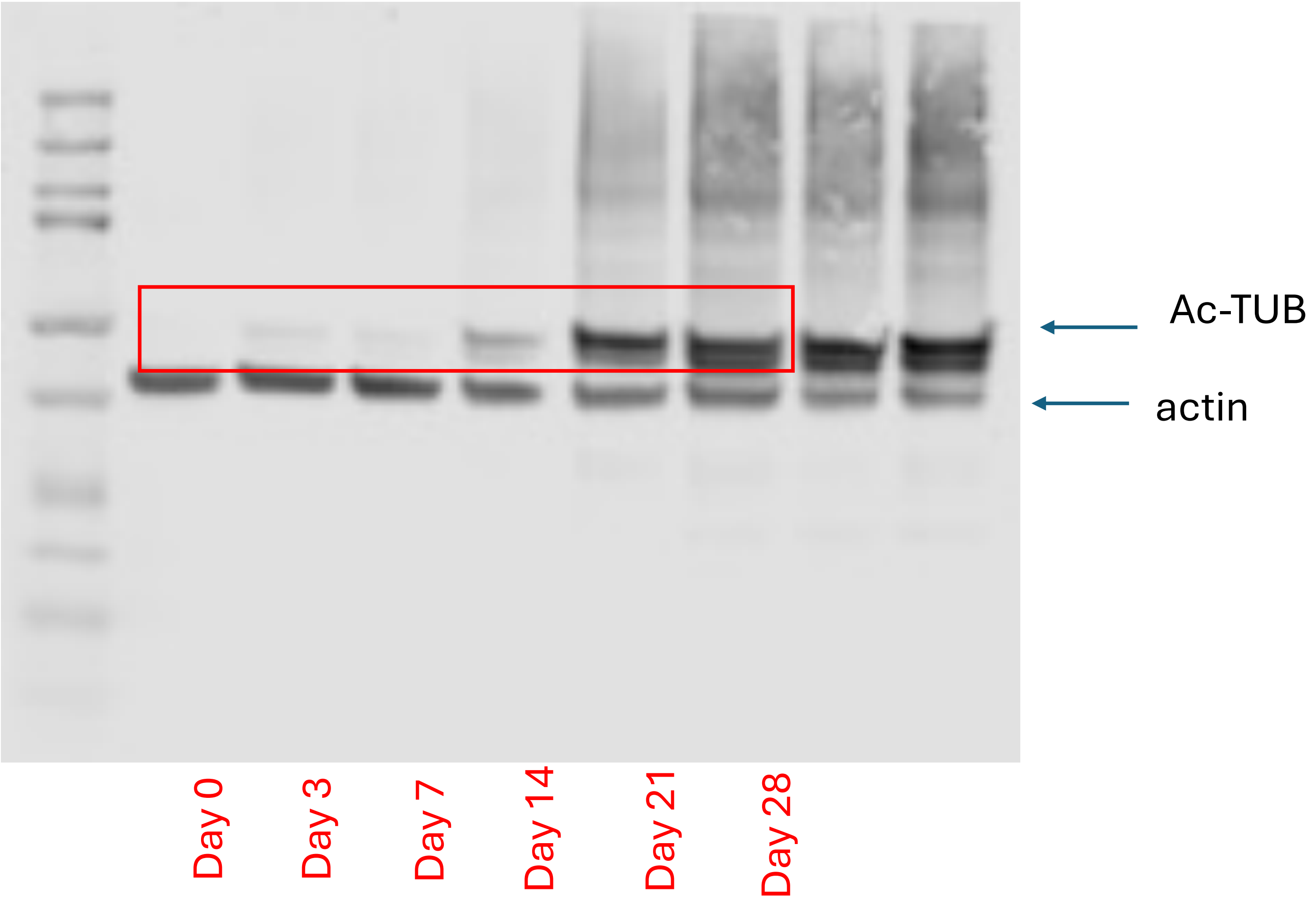

Full unedited gel for Figure 7E

actin lane

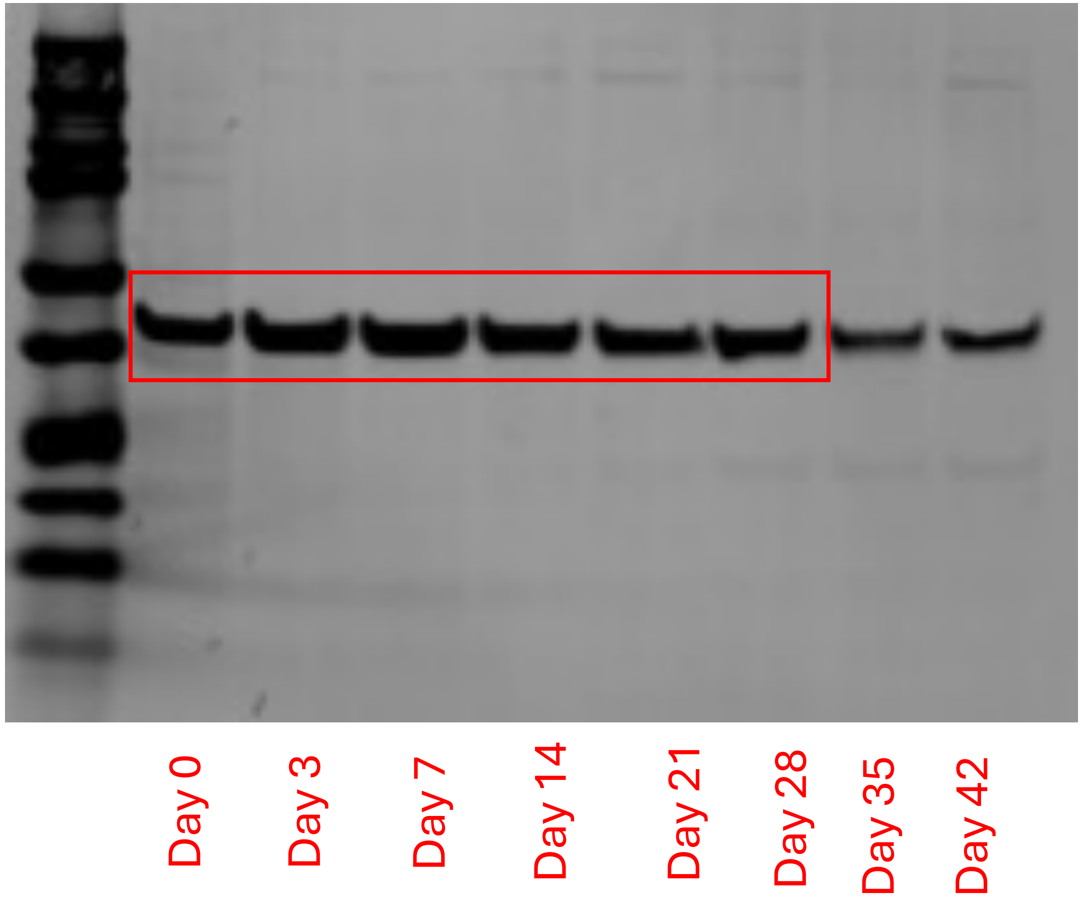

Supplement: Unedited blot and gel images [file jciinsight-9-180198-s200.pdf]
