# Supplementary material for: Transcriptional analysis of primary ciliary dyskinesia airway cells reveals a dedicated cilia glutathione pathway
Source: JCI Insight. 2024 Jul 23;9(17):e180198. doi: 10.1172/jci.insight.180198 (PMC11385084; doi:10.1172/jci.insight.180198)
Supplement: Supplemental table 6 [file jciinsight-9-180198-s205.docx]

**Supplemental Table 6.** *Chlamydomonas reinhardtii* isolated cilia analysis

| **Protein** | **Normalized average of**  **Wild-type**  **n = 3** | **Normalized average of**  ***oda1***  **n = 3** | **Normalized average of**  ***oda3***  **n = 3** | **Percent of Peptides in Membrane + Matrix (1)** |
| --- | --- | --- | --- | --- |
|  |  | DC2/ODAD1 | DC1/ODAD3 |  |
| **Outer dynein arm** | | | | |
| α-tubulin | 1 | 1.14 | 0.98 | 28 |
| β-Tubulin | 1 | 1.5 | 1.14 | 27 |
| **Outer arm docking complex** | | | | |
| DC1  Cre17.g703850 | 1 | 0.032 | 0.046 | 0 |
| DC2  Cre16.g666150 | 1 | 0.032 | 0.05 | 0 |
| DC3  Cre14.g617550 | 1 | 0 | 0.05 | 0 |
| **Outer dynein arms** | | | | |
| Dynein heavy chain alpha subunit (α-HC)  Cre03.g145127 | 1 | 0.033 | 0.048 | 25 |
| Dynein heavy chain beta subunit (β-HC)  Cre09.g403050 | 1 | 0.033 | 0.047 | 22 |
| Dynein heavy chain gamma subunit (γ-HC)  Cre11.g476050 | 1 | 0.033 | 0.044 | 20 |
| IC1  Cre12.g536550 | 1 | 0.043 | 0.058 | 17 |
| IC2  Cre12.g506000 | 1 | 0.033 | 0.08 | 4 |
| LC1  Cre02.g092850 | 1 | 0.033 | 0.054 | 17 |
| LC3  Cre12.g527750 | 1 | 0.037 | 0.044 | 6 |
| LC4  Cre01.g051250 | 1 | 0.071 | 0.088 | 0 |
| LC5  Cre17.g714250 | 1 | 0.06 | 0.12 | 18 |
| LC8*  Cre03.g181150 | 1 | 0.86 | 0.77 | 34 |
| LC9  Cre10.g428850 | 1 | 0.02 | 0.04 | 10 |
| LIS1  Cre12.g552900 | 1 | 0.09 | 0.12 | 60 |
| **Inner and outer dynein arms** | | | | |
| LC7a  Cre08.g376550 | 1 | 0.75 | 0.79 | ND |
| LC7b  Cre12.g546400 |  | 0.74 | 0.77 | 32 |
| **Redox proteins^1^** | | | | |
| GST (Alpha)  Cre16.g670973 | 0 | 0 | 0 | 73 |
| GST (Alpha)  Cre16.g682725 | 0 | 0 | 0 | 86 |
| GST (Alpha)  Cre16.g688550 | 0 | 0 | 0 | 1 |
| GST (Theta)  Cre17.g708300 | 0 | 0 | 0 | ND |
| GST (Theta)  Cre15.g636800 | 0 | 0 | 0 | 1 |
| GST (Theta)  Cre15.g636750 | 0 | 0 | 0 | ND |
| GST (Theta?)  Cre02.g142200 | 0 | 0 | 0 | ND |
| GST (Pi)  Cre17.g742300 | 1 | 1.2 | 1.4 | 94 |
| GST (Pi)  Cre17.g742450 | 0 | 0 | 0 | 74.5* |
| PRX2  Cre02.g114600^1^ | 1 | 1.52 | 0.81 | 72 |
| THX  Cre09.g391900^1^ | 1 | 1.2 | 0.83 | 1.0 |
| FAP102  Cre09.g394200^1^ | 1 | 1.77 | 2.62 | 55 |
| MST1  Cre16.g650600^1^ | 1 | 0.74 | 1.14 | 46 |
| AGG4  Cre17.g738050 | 1 | 1.58 | 1.69 | 67 |
| AGG6  Cre10.g456050 | 1 | 2.23 | 2.4 | 82 |
| **Inner dynein arm** | | | | |
| IC138  Cre12.g520950 | 1 | 1.14 | 1 | 17 |
| DHC2  Cre09.g392282 | 1 | 1.14 | 1.02 | 7 |
| **Membrane + Matrix proteins (Intraflagellar Transport proteins)** | | | | |
| IFT20  Cre02.g089950 | 1 | .58 | .63 | 13 |
| IFT52  Cre04.g219250 | 1 | 0.74 | 0.85 | 65 |
| IFT122  Cre01.g065822 | 1 | 0.76 | 0.84 | 81 |
| IFT144  Cre13.g572700 | 1 | .34 | .54 | 86 |

The proteins of isolated axonemes from wild-type as well as outer dynein docking complex mutants and analyzed in triplicate using mass spectroscopy. These preparations isolated axoneme and contained little of the Membrane + Matrix fraction. The protein name and the *Chlamydomonas* gene names are given. Wild-type was set to 1 and the log_2_ values was used to calculate the normalized ratio. LC2 (Cre12.g527750) and LC6 (Cre03.g187200) were not present in the wild-type control data. Data for inner dynein arm and IFT proteins are included as controls for axonemal proteins and membrane/matrix proteins, respectively.

Membrane /Matrix / Axonemal ratio generated from gametic cells (1) for which the axonemal fraction and the Membrane + Matrix fraction were both isolated. For Cre17.g742450, the ratio in vegetative cells is shown.

*LC8 is found in the radial spokes and inner dynein arms as well as the outer dynein arms

^1.^ Proteins that show formation of disulfide bonds in response to changes in redox poise (2).

1. Sakato-Antoku M, and King SM. Developmental changes in ciliary composition during gametogenesis in Chlamydomonas. *Mol Biol Cell.* 2022;33(7):br10.

2. Wakabayashi K, and King SM. Modulation of Chlamydomonas reinhardtii flagellar motility by redox poise. *J Cell Biol.* 2006;173(5):743-54.
